# Single-photon avalanche diode imagers in biophotonics: review and outlook

**DOI:** 10.1038/s41377-019-0191-5

**Published:** 2019-09-18

**Authors:** Claudio Bruschini, Harald Homulle, Ivan Michel Antolovic, Samuel Burri, Edoardo Charbon

**Affiliations:** 1 AQUA, EPFL, Neuchâtel, Switzerland; 2 AQUA, TU Delft, Delft, The Netherlands

**Keywords:** Biophotonics, Imaging and sensing

## Abstract

Single-photon avalanche diode (SPAD) arrays are solid-state detectors that offer imaging capabilities at the level of individual photons, with unparalleled photon counting and time-resolved performance. This fascinating technology has progressed at a very fast pace in the past 15 years, since its inception in standard CMOS technology in 2003. A host of architectures have been investigated, ranging from simpler implementations, based solely on off-chip data processing, to progressively “smarter” sensors including on-chip, or even pixel level, time-stamping and processing capabilities. As the technology has matured, a range of biophotonics applications have been explored, including (endoscopic) FLIM, (multibeam multiphoton) FLIM-FRET, SPIM-FCS, super-resolution microscopy, time-resolved Raman spectroscopy, NIROT and PET. We will review some representative sensors and their corresponding applications, including the most relevant challenges faced by chip designers and end-users. Finally, we will provide an outlook on the future of this fascinating technology.

## Monitoring progress of single light particle detectors for biophotonics applications

Substantial improvements have been made in the past 15 years to imagers based on a device that acts like a 3-in-1 light particle detector, counter and stopwatch, furthering their potential use in biological imaging technologies. Claudio Bruschini of Switzerland’s École polytechnique fédérale de Lausanne and colleagues reviewed the developments in the use of single-photon avalanche diode (SPAD) arrays for biophotonics applications. They found that, while most SPAD imagers are still used in specialised research settings, significant improvements have been made to their sensitivity, reliability and reproducibility, and a host of sensor architectures have been explored. When a photon, the unit of light, hits a SPAD, it triggers an almost immediate electric current. Compact SPAD arrays implemented in standard CMOS electronic circuits can thus be used, for example, to measure in parallel the fluorescence lifetime of molecules tagged onto living cells and tissues, improving our ability to observe them. The review was completed by an outlook of this fascinating technology and an analysis of the most relevant challenges still lying ahead.

## Introduction

Individual single-photon avalanche diodes (SPADs) have long been the detector of choice when deep sub-nanosecond timing performance is required, due to their excellent single-photon detection and time-stamping capability^1–4^. The breakthrough implementation of the first SPADs in standard complementary-metal-oxide semiconductor (CMOS) technology^5^ triggered the exploration and design of large digital SPAD imagers, potentially manufactured in volume at affordable prices. This was soon followed by the first integrated SPAD array^6^ and a host of architectures, ranging from simpler implementations of the early days, based solely on off-chip data processing, to progressively “smarter” sensors including on-chip, or even pixel level, time-stamping and processing capabilities. Modular setups have also been designed, either through the combination of SPAD arrays with FPGAs (“reconfigurable pixels”), or by means of very recent 3D developments. Furthermore, basically all implementations rely on FPGA-based host boards; combined with the natively digital SPAD data output, this opens the door to real-time algorithmic implementations in close proximity to the sensor, such as FPGA-based autocorrelation and lifetime calculations.

As SPAD technology matured, a range of applications have been explored in very diverse fields, such as consumer and robotics imaging, data and telecom security, advanced driver-assistance systems and biophotonics, which is the main subject of this review. In particular, we will discuss (endoscopic) fluorescence lifetime imaging (FLIM), (multibeam multiphoton) FLIM-FRET (Förster resonance energy transfer), single-plane illumination fluorescence correlation spectroscopy (SPIM-FCS), localisation- and entangled photons-based super-resolution microscopy (SRM), time-resolved Raman spectroscopy, near-infra-red optical tomography (NIROT) and positron emission tomography (PET). However, it is true that SPAD imagers are still mostly used in specialised research settings, apart from some notable non-imaging exceptions, such as SPAD arrays in the form of silicon photomultipliers (SiPMs), which are readily available from a number of manufacturers. This is at first glance surprising, given the aforementioned potential for unrivalled photon counting and time-resolved performance; however, it can be partly traced back to some performance parameters that still lag behind those of established CCDs and sCMOS imagers, such as the quantum efficiency over the whole spectrum and the fill factor, which are of importance for several light-starved applications. The pixel sizes are typically larger, limiting so far the manufacturing of megapixel arrays. On the technological side, the design of high performance, low-noise SPADs is challenging; the same is true at the system level for data handling, leading to important firmware development efforts. Therefore, it is not surprising that recent efforts have been focused, at the device level, on increasing the SPADs’ key figures of merit^7^ and improving the contact with foundries, to fully profit from possible process optimisations.

In the following sections, which are mostly dedicated to SPAD imagers in standard CMOS technologies, we will first discuss the SPAD state-of-the-art, starting from individual devices, their key properties and the corresponding optimisation trade-offs, which are strongly influenced by the target application. We will then focus on the impact of design choices on the overall sensor architecture and the most important challenges, moving up in a vertical fashion from the pixel level, considering the basic circuitries and in-pixel options, to array architectures (1D vs. 2D) and the read-out, which is of particular importance for real-time implementations. A host of biophotonics applications will then be described, starting from FLIM and its different flavours and ending with more disruptive scenarios and sensor concepts such as quantum-based super-resolution microscopy and 3D-stacking (the combination of a top sensor layer with a bottom control and processing layer), respectively.

Interested readers are encouraged to refer to ref. ^3^ for details of other applications of SPAD-based imagers and to refs. ^8–11^ for a comparison with established devices and alternative CMOS imagers.

## SPAD detectors and imagers

### Single-photon avalanche diodes

**Fig. 1:**
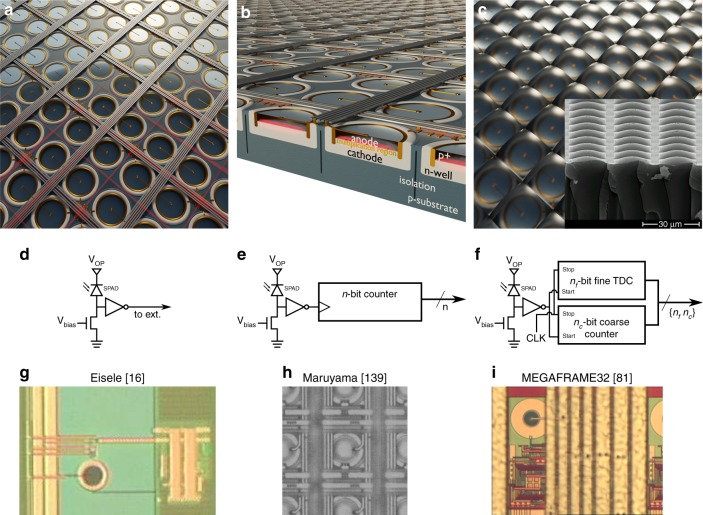
SPAD arrays and comparison of the SPAD pixel architectures. **a** Artist’s impression of a SPAD array (top view) and **b** an example of the corresponding cross-section for a substrate isolated SPAD in a conventional CMOS process, depicting some of the key components (diode anode/cathode and corresponding p-n junction, multiplication region in which the avalanche is triggered, and the substrate and isolation from it)^3^. The SPAD fill factor can be enhanced with microlenses (**c**), and the inset shows an SEM image from ref. ^15^. The design of individual pixels ranges from **d** basic structures, which are only capable of generating digital pulses corresponding to individual photon arrivals on the SPAD, to **e** pixels including counters, which add the individual arrivals over a given time window that is possibly gated, or **f** more advanced electronics such as a complete TDC, which make it possible to time-stamp individual photon arrival times. The corresponding examples of pixel micrographs are displayed in **g**–**i**, as reprinted from refs. ^16,81,139^

Photodetectors capable of measuring single photons have been known for decades and have been realised using different technologies, from photomultiplier tubes (PMTs) to microchannel plates (MCPs) and electron-multiplying charge-coupled devices (EMCCDs). However, the implementation of large, all solid-state single-photon imagers (Fig. 1a) calls for a new kind of miniaturised, scalable device featuring a reliable set of performance parameters. One example of such a device is represented by the SPAD implemented in industry-standard CMOS technology. The SPAD is basically a photodiode whose p-n junction (Fig. 1b) is reverse biased above its breakdown voltage *V*_bd_, such that a single photon incident on the active (i.e. photosensitive) device area can create an electron-hole pair and thus trigger an avalanche of secondary carriers. The avalanche build-up time is typically on the order of picoseconds, so that the associated change in voltage can be used to precisely measure the time of the photon arrival^1,12^. This operation regime is known as Geiger mode; hence, the devices are also known as Geiger-mode APDs (GmAPDs).

The self-sustaining avalanche in the SPAD needs to be stopped as soon as possible to prevent the destruction of the device itself due to the high current. The corresponding quenching occurs by lowering the SPAD bias voltage *V*_OP_ below the breakdown voltage, e.g. by using a resistor in series with the SPAD. The voltage across the SPAD then needs to be restored to its initial value above the breakdown voltage, before the next photon can trigger another avalanche. During this interval, which is typically on the order of tens of nanoseconds and known as dead time, the SPAD will be almost insensitive, depending on the exact quenching and recharge mechanism and SPAD front-end implementation. The sensitivity of the SPAD will gradually increase, until it reaches its nominal value when the recharge is complete. The change in voltage across the SPAD during a detection event is easily transformed into a digital signal by using a front-end discriminator, for example a single transistor or an inverter; the discriminator has an important role in determining the minimum detectable avalanche current. The resulting output, which does not depend on the wavelength of the impinging photon, is compatible with standard electronics, which makes it easy to integrate a SPAD into larger circuits and arrays of detectors. Table 1 summarises the most important properties of SPADs and compares them across the SPAD-based imagers reported in Table 2.

**Table 1:** Key SPAD pixel parameters and typical values commonly found in the sensors listed in Table 2

	Value range
SPAD pixel	
Dead time [ns]	10–100
DCR [cps/μm^2^]	0.3–100
PDP (peak) [%]	10–50
Fill factor [%]	1–60
Timing resolution [ps]	30–100
Afterpulsing probability [%]	0.1–10

**Table 2:** Overview of standard CMOS SPAD imagers targeting biophotonics applications, in chronological order, as published over the past 15 years

Sensor and architecture	Year	SPAD array	Technology [nm]	SPAD diameter_eq._ [μm]	Pixel pitch [μm]	Fill factor [%]	PDE_top_ [%]	DCR [cps/μm^2^]	Timing technique	Sensor specifications	System features	Application
First CMOS SPAD array^6^	2003	8 × 4	800	6.4	—	<1	0.2	1.6	—	—		—
Rech^112–116,118,119^	2007	8 × 1	—	50.0	198	5	2.5	1.0	—	—		FRET/FCS
Schwartz^79,80^	2007	64 × 64	350^HV^	4.1	40	<1	0.1	71.0	TCSPC + gating	In-pixel TDC	4096 in-pixel 350 ps 10b TDCs	FLIM
Niclass (LASP)^17,41,151^	2008	128 × 128	350^HV^	7.0	25	6, × 2−8^ml^	2.1, × 2−8^ml^	17.0	TCSPC	Column-based TDCs	32 column 98 ps 10b TDCs	NIROT
Boiko (G^(2)^)^161,162^	2009	4 × 4	350^HV^	3.5	36	<1	—	1.0	—	—		
Niclass (FluoCAM)^26,31,60^	2009	60 × 48	350^HV^	8.6	85	<1	0.1	7.0	Gating (2 × )	2 in-pixel 8b counters	5 ns gate, 12 ps steps	FLIM
Guerrieri^93,102–104^	2009	32 × 32	350^HV^	20.0	100	3.1	1.3	12.7	Gating	In-pixel 8b counter		FLIM/FCS
MEGAFRAME32^21,50,51,81,82,85,86,89–92,105^	2009	32 × 32	130^CIS^	5.6	50	1	0.4	4.0	TCSPC	In-pixel TDC	1024 in-pixel 50 ps 10b TDCs	FLIM/FCS/FRET
Pancheri^18^	2009	64 × 4	350^HV^	17.6	26	34	10.9	4.3	Gating (4×)	4 in-pixel 8b counters	4 SPADs = 1 pixel	FLIM
Carrara (RadHard2)^106,107,156,157^	2009	32 × 32	350^HV^	6.0	30	3.1	1.1	5.0	–	In-pixel 1b counter		FCS/NIROT
Stoppa^19^	2009	7 × 2	350^HV^	—	—	—	—	13.0	Gating	In-pixel 17b counter		FLIM
Maruyama^20,140^	2011	128 × 128	350^HV^	6.0	25	4.5, × 1.6^ml^	0.9, × 1.6^ml^	6.6	Gating	In-pixel 1b counter		FLIM/Raman
MEGAFRAME128^33,83,84^	2011	160 × 128	130^CIS^	5.6	50	1	0.3	2.0	TCSPC	In-pixel TDC	20480 in-pixel 55 ps 10b TDCs	FLIM
Pancheri^76–78^	2011	32 × 32	350^HV^	12.9	25	20.3	—	5.4	Gating	In-pixel analogue counter	1.9 ns gate	FLIM
Durini (BackSPAD)^168,169^	2012	32 × 32	350^3D^	94.4	50	75.4	—	39.7	—	In-pixel counters	Preliminary	
Tyndall^62–64^	2012	32 × 32	130^CIS^	8.0	22	10	—	13.7	TCSPC	Per group TDC	16 52 ps 16b TDCs, mini-SiPM	FLIM
Field^34,35^	2013	64 × 64	130	5.0	48	<1	0.3	28.0	TCSPC	Column-based TDCs	4096 column 62.5 ps 10b TDCs	FLIM
Mandai^22^	2013	416 × 4 × 4	350^HV^	32.6	30/50	55.6	17.0	39.0	Majority time voting	Column-based per group TDC + in-pixel 1b counter	192 column 44 ps 17b TDCs	PET
Maruyama^139,141^	2013	1024 × 8	350^HV^	18.0	24	44.3	9.6	29.0	Gating	In-pixel 1b counter	0.7 ns gate, 250 ps steps	Raman
Nissinen^138,142,143,145^	2013	128 × 8	350^HV^	9.7	33	23	5.8	71.0	Gating (4×)	4-pixel gate comparators	4 SPADs = 1 pixel	Raman
Walker (SPADnet1)^48,49,163,164^	2013	720 × 16 × 8	130^CIS^	16.3	19	42.9	12.0	6.2	Majority time voting	In-pixel TDC + 7b counter	128 in-pixel 64 ps 12b TDCs + histogram generation	PET
Burri (SwissSPAD)^15,44,75,125,129,178^	2014	512 × 128	350^HV^	6.0	24	5, ×8−12^ml^	2.3, ×8−12^ml^	12.0	Gating	In-pixel 1b counter	4 ns gate, 20 ps steps	FLIM/FCS/SRM
Carimatto^23^	2015	416 × 18 × 9	350^HV^	33.0	30/50	57	18.6	43.0	Majority time voting	Column-based per group TDC + in-pixel 1b counter	432 column 48 ps 17b TDCs	PET
Krstajic′^24,67^	2015	256 × 8	130^CIS^	18.2	24	43.7	—	5.4	TCSPC + gating	Per-pixel TDC + histograms	512 per-pixel 40 ps TDCs + histogram generation	FLIM/Raman
Parmesan^37^	2015	256 × 256	130^CIS^	4.2	8	19.6	—	4.0	TCSPC	TAC pixels	External 14b ADC	FLIM
Mata Pavia (3DAPS)^152,167^	2015	400 × 1	130^3D^	6.0	11	23.3	2.8	357.0	TCSPC	In-pixel TDC	3D stacked, 50 ps 12b TDCs	NIROT
Abbas^173^	2016	128 × 120	65^3D^	5.9	8	45	12.4	36.2	Gating	In-pixel 12b counter	3D stacked, 65 nm top-tier/45 nm bottom-tier	
Lee^32^	2016	72 × 60	180	15.0	35	14.4	0.4	2.3	Gating	In-pixel 10b counter	10 ns gate, 72 ps steps	FLIM
Burri (LinoSPAD)^54,55^	2016	256 × 1	350^HV^	17.1	24	40	13.6	11.0	TCSPC (External)	—	64 FPGA-based 25 ps TDCs	FLIM/Raman
Perenzoni^38^	2016	160 × 120	350^HV^	7.8	15	21	—	12.0	Gating	Column analogue counter	10 ns gate, 194 ps steps	FLIM
Dutton (SPCIMAGER)^25,45,130^	2016	320 × 240	130^CIS^	4.7	8/16	26.8, × 1.8−2^ml^	10.6, × 1.8−2^ml^	3.0	Gating	In-pixel analogue counter		FLIM/SRM
Erdogan^69^	2017	1024 × 16	130^CIS^	18.8	24	49.3	—	—	TCSPC + gating	Per-pixel TDC + histograms	512 per-pixel 50 ps TDCs + histogram generation	FLIM
Holma^146,148^	2017	256 × 16	350^HV^	18.0	35	26	—	—	TCSPC	Shared TDCs	Two 52 ps 3b TDCs	Raman
Kufcsák^68^	2017	256 × 8	130^CIS^	18.2	24	43.7	—	5.4	TCSPC + gating	Per-pixel TDC + histograms	Improvement of^24^	FLIM/FRET/Raman
Lindner (Piccolo)^46,95,153,154^	2017	32 × 32	180	17.0	28	28	13.4	0.6	TCSPC	Column-based TDCs, dynamic reallocation	128 column 49 ps TDCs	NIROT
Ulku (SwissSPAD2)^29,73,74^	2017	512 × 512	180	6.0	16	10.5	5.2	0.3	Gating	In-pixel 1b counter	5 ns gate	FLIM
Gyongy^30^	2018	256 × 256	130^CIS^	14.1	16	61	—	51.0	Gating	In-pixel 1b counter		FLIM

All values and operating modes are reported as listed in the literature

*SPAD diameter*_*eq*_ $2\sqrt {{\mathrm{SPAD}} \ {\mathrm{area}}/\pi}$, *PDE* SPAD photon detection probability at the nominal excess bias voltage, multiplied by the pixel fill factor, *DCR* median (or average if not indicated) dark count rate per SPAD unit area, for the same excess bias voltage as the PDE

Operating mode definitions: *TCSPC* time-correlated single-photon counting, *Gating* use of one or multiple moving gates, *Majority time voting* generation of a time-stamp per event (on the first arrived photon in a pixel, in the simplest case), only if a certain photon count is reached

^ml^Use of microlenses—the quoted native PDE/fill factor needs to be multiplied by a concentration factor

^CIS^CMOS imaging sensor process

^HV^CMOS high-voltage process

^3D^3D integration technology (usually backside illuminated)

A number of parameters are used to describe the performance of a single SPAD device^7^. The most important is the photon detection probability (PDP), which represents the avalanche probability of the device in response to photon absorption at a given wavelength. In CMOS SPADs, the PDP has a peak in the visible region, which can reach 70% for single, optimised diodes^13^. Other important parameters are the dark count rate (DCR), i.e. the observed avalanche rate in the absence of light, and afterpulsing, which introduces false events that are correlated in time with previous detections^7^. When SPADs are grouped in imagers one must consider electrical and optical crosstalk and the fill factor, which denotes the ratio between the photosensitive area and the total pixel area. Although the fill factor is generally calculated from the drawn area, the actual fill factor might be slightly lower, typically by a few percentage points, due to edge effects, leading to an inactive SPAD area (see ref. ^14^ for a detailed discussion of the so-called inactive distance). The SPAD’s fill factor obviously affects the overall imager sensitivity, given that it is multiplied by the PDP to give the overall photon detection efficiency (PDE).

Many of the SPAD characteristics can be optimised in the design phase, often requiring trade-offs. For example, a larger size of the guard ring, to better contain the high electric field and prevent a premature edge breakdown, will positively impact the crosstalk, at the expense of the active area and thus the fill factor. This can be compensated with larger diodes at the cost of the DCR, which increases with diode area. A short dead time allows a higher count rate, and thus a high dynamic range, but increases the afterpulsing probability, which leads to problems when detecting photon correlations. The targeted sensor application should ideally be taken into account during the design phase to select the optimal trade-offs, such as sensitivity versus noise and speed versus fill factor.

### From individual SPADs to arrays

When a suitable SPAD device and pixel circuit have been demonstrated in a given fabrication process, they can be integrated into an array to form a SPAD imager. The simplest array is linear, allowing the designer to place the detection and processing electronics outside the photosensitive area, thus achieving higher fill factors. A 2D array of pixels, on the other hand, requires self-contained circuits, in-pixel or at the periphery, to acquire, store and transmit data. This additional circuitry negatively impacts the fill factor, but eliminates the need for scanning to create a complete image. Some freedom also exists at the level of the spatial granularity; grouping pixels, for example, reduces the overall data throughput, while preserving key information, such as photon timing and reducing the complexity. The same is true for the temporal granularity, allowing, for example, the acquisition of only a subset of all possible timestamps for specific applications. Finally, the sensor fabrication itself might include post-processing steps, such as the deposition of microlenses to increase the overall sensitivity (Fig. 1c).

We discuss the various architectural choices and the corresponding trade-offs below, moving from the pixel level up to the array design specificities.

## Architectures

### Pixel architecture

We divide SPAD pixel circuits into three broad types, depending on the functionality added on top of the basic photon-to-electrical pulse conversion. The first type is represented by a basic structure, which only includes the circuitry necessary for a full detection cycle consisting of the avalanche generation, quench and recharge. The output of such a pixel is a train of electrical pulses corresponding to individual photon detections. The second type is a pixel with built-in counter, consisting of a counting circuit and at least one bit of memory; its output is a photon count. The third type of pixel is time-correlated and includes circuitry to discriminate the arrival time of photons; its output can be as simple as a flag for a detection during a given time window or as complex as a variable number of timestamps reporting distinct photon arrival times. Concept schematics for the three types of pixels are shown in Fig. 1d–f, while selected implementation examples are displayed in Fig. 1g–i. The pixel fill factor obtained when assembling an array is inversely proportional to the amount of electronics placed besides the SPAD, which makes it advantageous to use modern fabrication technologies that enable smaller feature sizes.

The pixel design elements common to all types of pixels include active quenching and recharging, masking and gating. Active quenching and recharging can be employed to optimise the detection cycle of a SPAD by reducing the dead time in a well-controlled manner and thus improving the maximum count rate. In this case, active circuitry can be used to stop the avalanche and recharge it earlier than what is possible with passive resistive approaches. This limits the amount of charges flowing through the diode, improving its lifetime and reducing the afterpulsing. Active techniques for quenching and recharging have been employed in a number of designs^12,15–26^.

It is worth noting that a measured SPAD count actually corresponds to an event with one or more simultaneously detected photons, whose exact number cannot be resolved (SPADs are indeed also termed click detectors^27^). Nevertheless, one can still estimate the number of detected photons by using preknowledge about the actual SPAD quenching and recharging mechanism and the temporal distribution, e.g. exponential, of the impinging photons^28^.

Masking is used to selectively disable pixels inside an array. This feature is commonly employed in designs where multiple pixels share circuitry to avoid overloading by particularly noisy pixels, which would otherwise decrease the overall performance. Possible implementations can either switch off the SPAD, thereby preventing avalanches from taking place, or disconnect pixels from the read-out circuit. Switching off the SPAD has the additional benefit of removing possible crosstalk. Examples of pixel architectures with masking are^20,22–24^.

Gating is another independent element of pixel architectures and consists of enabling the SPAD only for a limited time, down to picosecond windows. Gating can be applied directly to the SPAD (device gating) or to its front-end electronics (electrical gating). In the case of SPAD gating, either the cathode bias needs to be lowered below the breakdown voltage, or the anode bias needs to be increased above the excess bias; the observation window starts when the SPAD is activated. If the required bias swing is higher than what is applicable in standard CMOS circuitry, external bias control is needed. In this case, however, the gate’s rise and fall times are increased due to the capacitance of the external gating line. In contrast, gating the front-end electronics can be directly integrated into the CMOS circuitry, thus enabling sharper gate profiles. The drawback is that the SPAD is still active outside the gate, and has, therefore, to be recharged before opening the gate.

The key advantage of gating is the temporal discrimination that can be achieved through its implementation, even though the overall detection efficiency is reduced. In a setup with repetitive (pulsed) illumination, gating permits the selective capture of photons during a portion of the repetition period, with the added option of shifting the gating window in picosecond steps. This feature can also be used to exclude parts of the photon response that are not of interest. Exemplary gating applications include rapid lifetime determination in FLIM, the accurate reconstruction of a particular optical response in the time domain, the elimination of early/background-related detections, or the reduction of a sample’s intrinsic fluorescence in Raman spectroscopy. Gating can also be used to reduce the DCR by eliminating dark counts occurring outside the time zone of interest.

Moving beyond the basic structures, pixels have been designed to include memory elements for multiple purposes. A single-bit memory can be used to capture a purely binary image during a given time interval (i.e. a frame time), for example when it is important to avoid global shutter artefacts; this kind of architecture can be implemented with only a few transistors, thus still allowing for reasonable native fill factors (5–10%), while a fast read-out (10–100 kpfs) is usually employed to increase the dynamic range and accumulate multi-bit images off-chip^15,20,29^. Fabricated in a 130 nm CMOS process, the pixel in ref. ^30^ reaches a notable fill factor of 61% by using an all NMOS design, an analogue storage element, and deep *n*-well sharing between the pairs of SPAD rows, at the expense of a reduction in the timing accuracy due to the simplified gating circuit, and a somewhat increased crosstalk between pixels.

Multi-bit and multiple counters allow differentiation between the number of captured photons. When used together with multiple gates, a simple in-pixel phase detector can be constructed and read-out requirements can be relaxed, while maintaining a good dynamic range. Integrating more memory in each pixel drastically reduces the fill factor and makes it advantageous to move to smaller technology nodes. For example, the 2 × 8-bit counter pixels detailed in ref. ^31^, implemented in older 0.35 μm technology, result in a fill factor of 0.8%, while the 10-bit counter pixel reported in ref. ^32^, designed with a 0.18 μm process, results in a fill factor of 14.4%.

Pixels with an integrated arrival time measurement, typically implementing time-to-digital converters (TDCs) or their analogue counterparts (time-to-analogue converters or TACs), represent the most powerful, but also the most complex pixel architecture. The timing circuitry in general needs to be as compact and low power as possible to be integrated in every pixel of an imager, while still offering the required timing resolution. Arrays with in-pixel TDCs usually do not exceed fill factors of a few percent^21,33–35^, with ref. ^36^ representing a notable exception, reaching a fill factor of over 19%. A ring-oscillator is typically used for time-stamping with a resolution of tens of picoseconds (fine measurement), whereas the timing range is extended with a counter as needed (coarse measurement). Analogue techniques, such as in-pixel or column-level TACs or analogue memories in the form of capacitors, are making a comeback because they can be implemented in area-efficient ways, at the expense of analogue-to-digital converters placed at the periphery of the array or outside of the chip, and the difficulties inherent in mixed-signal design, e.g. non-uniformities and mismatch. Notable examples of sensors using analogue elements are refs. ^4,25,37,38^, the last of which presents an array with a fill factor of 26.8%.

### Array architecture

**Fig. 2:**
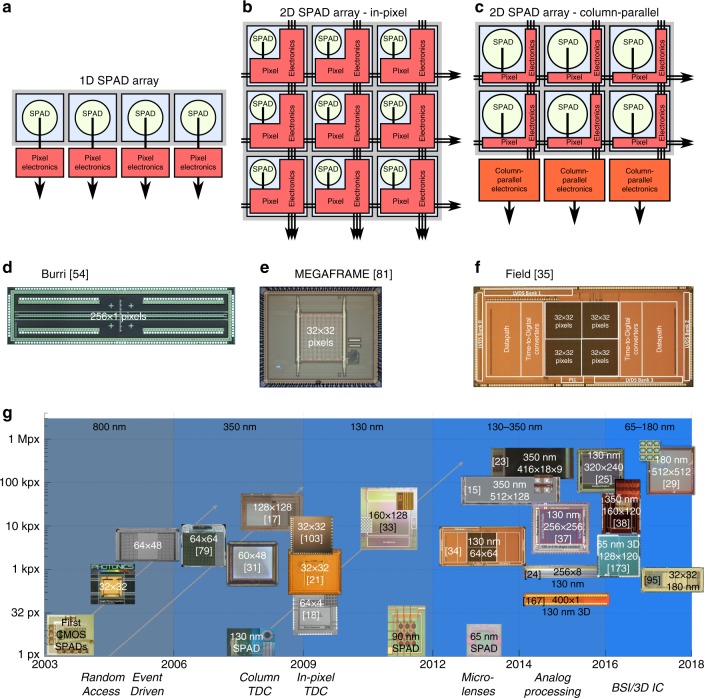
Comparison of the SPAD array architectures. **a** In linear arrays, the pixel electronics can be placed outside the pixel area, leading to an increase in the fill factor; in 2D arrays, the fill factors are generally smaller, because **b** electronics is needed inside the pixel itself, or at least **c** at the array periphery, e.g. for column-based TDCs. The related advantages and disadvantages are discussed in detail in the text, and the corresponding examples of array micrographs can be found in **d**–**f**, as reprinted from refs. ^35,54,81^. Finally, **g** provides an overview of the evolution of SPAD imagers over the last 15 years in terms of the total number of pixels (on the vertical axis), the technology node (indicated at the top of the image), and some salient architectural characteristics, such as random access or event driven (indicated at the bottom of the image). Only some representative examples, primarily targeted at biophotonics applications, are shown here (details are reported in Table 2). The diagonal lines indicate the developments along a given technology node (800, 350 and 130 nm), which are usually started by optimising the SPADs before designing full imagers. Recent years have seen a trend towards higher spatial resolutions and 3D IC solutions

The simplest form of a SPAD pixel array is a single line. In a linear (1D) array (Fig. 2a, d) all pixel electronics is placed outside the sensor area, with only the diode guard ring separating the active area of different pixels. Most linear SPAD arrays allow a truly parallel pixel operation, even if resource sharing is in principle possible in the same way as for 2D arrays. The 1D architecture allows to reach the highest possible fill factors, at the expense of the optical or mechanical scanning solutions that are needed to generate a 2D image, should this be required by the target application.

Two-dimensional SPAD pixel arrays (Fig. 2b, e) are capable of acquiring 2D images directly, at the expense of a more complex sensor design for the interconnection between pixels and read-out electronics. In general, all supply, control and data signals are shared across the rows and columns of a 2D pixel array to maximise the fill factor. The minimal circuitry needed at the pixel level is a read-out line driver, but usually more circuitry is added, such as gating and counters with memory, as discussed in the previous subsection. More complex pixels include time-stamping electronics and in-pixel photon information counting or timing pre-processing. Depending on the application requirements, some circuit elements, such as complex time-to-digital converters, can be shared among multiple pixels, either for a larger block of pixels or, more commonly, for (multiple) rows or columns (Fig. 2c, f). Non-uniformities and timing skews grow in general with increasing array size, and similarly the overall generated data volume also increases, calling for specific read-out solutions as discussed below. One possibility is to bin the pixels in groups, e.g. in situations where the spatial resolution can be traded off with the signal-to-noise ratio (SNR).

Despite the efforts to maximise the fill factors in 2D arrays, the obtained fill factors are usually below those of similarly sized sCMOS cameras (also see Table 2), especially for complex pixel architectures like in-pixel TDCs, due to the larger transistor counts. Microlenses, therefore, represent a viable option to reclaim some of the fill factor lost due to the electronics. These micro-optical devices are placed in front of the sensitive area, typically on the surface of the detector, and concentrate impinging photons onto the active (i.e. photosensitive) pixel area (Fig. 1c). Examples of SPAD-related microlens developments and sensors are presented in refs. ^20,39–45^. The microlenses are typically optimised for specific applications and properties (for example collimation) of the impinging light.

In 2D imagers, it is possible that the pixels no longer strictly operate in parallel, for example when they contain memory elements that are addressed and then reset by the read-out (to gather new photons) on a row-by-row basis (rolling shutter acquisition operation). This can lead to well-known artefacts, such as temporal lag, when recording phenomena with very fast dynamics. Therefore, some imagers implement a true global shutter operation, which provides an image snapshot at a given instant. This can be achieved by activating all pixels together at the start of a frame and then “freezing” the data acquisition at the end of the frame and starting the read-out operation, with some loss in efficiency (reduced temporal aperture). An alternative that does not call for (many) expensive global signals is represented by an event-driven operation mode, which allows continuous on the fly recording of events as they occur; one way of implementing this operation mode is by using a common bus shared by all pixels (e.g. in a column), with separate address lines to identify the SPAD that has fired^46^.

Considering all the trade-offs, encountered when selecting a pixel and array architecture, there is no single best implementation. The architecture of a SPAD array should, therefore, be chosen based on the target application, sometimes even abandoning classical imaging approaches, or at least benefiting from the flexibility provided by SPAD arrays, e.g. by binning pixels and pre-processing data close to the sensor. As an example, we consider time-of-flight positron emission tomography (ToF-PET), where the information of interest is represented by the energy, time-of-arrival and interaction coordinates of gamma rays; the gamma rays are converted by means of scintillating crystals into visible light photons, to be detected by SPAD arrays in the form of SiPMs. In this case, it makes sense to reduce the effective granularity of the recorded data by grouping multiple SPADs together and compensate for noisy detectors using masking. The gamma ray energy is given, in this digital approach, by the total number of SPADs that have fired in a time window of a few hundred nanoseconds, while the time-of-arrival can be estimated on chip and refined by the local control and communication FPGA. An overview of the digital approaches to SPAD-based sensors for PET is provided in ref. ^47^, while individual detector architectures are detailed in refs. ^22,23,48,49^.

### Read-out architecture

One of the main concerns when interfacing with a single-photon camera is the resulting high data rate, especially when recording timestamps of individual photons or working at very high frame rates. Eventually, the data rate needs to be reduced to a level where it can be transferred to a computer or storage medium. This can be realised, for example, with the same approach as what is used in a streak camera, whereby information captured during a (very) short duration is stored locally (in the pixel) at a high speed, and then read out at a low speed for processing and storage. Another possibility is represented by in situ extraction of higher level information. The corresponding algorithms, such as histogram accumulation or multi-bit count integration, can be implemented on the control FPGA, or even on the sensor itself. In the case of fluorescence lifetime imaging, for example, real-time systems have been devised that can calculate the lifetime of molecules at video rate, without the need for recording the full single-photon data stream^9,50,51^, including multi-exponential scenarios^52^. An FPGA system indeed offers some flexibility in terms of possible data processing and a high computational bandwidth, which can be used, for example, to realise firmware-based 32 × 32 autocorrelator arrays as detailed in ref. ^53^ (with FCS as the target application). The “reconfigurable pixel” concept proposed in refs. ^54,55^ maximises flexibility by moving the whole circuitry, which is usually placed beyond the basic SPAD pixel structure, inside the FPGA; this makes it possible to implement different TDC or counter architectures, with the goal of tailoring the system (sensor and firmware) in an optimal way according to the needs of the target application.

### SPAD sensor summary

Table 2 lists a comprehensive summary of the main SPAD-based sensors and imagers that have been designed for biophotonics applications; they are discussed in the next section, together with the related applications and the corresponding results. A representative subset is shown in Fig. 2g, which provides a graphical overview of how these imagers have evolved over the last 15 years.

## Biophotonics applications

The following sections analyse in detail a host of biophotonics applications that have been explored with SPAD imagers, starting from FLIM, which has been addressed early on, and its different flavours, ending with more disruptive scenarios and forward looking sensor concepts. The use of SPADs in these applications is summarised in Table 3 and compared to the use of non-SPAD-based methods, highlighting the SPAD benefits and drawbacks, ongoing technology developments and selected experiments.

**Table 3:** Overview of the main biophotonics applications that have been explored with standard CMOS SPAD imagers, their conventional counterparts, advantages and disadvantages, selected experimental highlights and the predicted direction of further developments

Application with key review papers	Non-SPAD methods/sensors	SPAD array architecture	SPAD advantages/disadvantages	Experimental highlights	Technology development directions
FLIM^9,10,56,66,94^	PMT, hybrid, APD	point-like, linear	+ Increased count rate due to pixel parallelisation, on-chip histogram generation and/or lifetime estimation − DCR, sensitivity, system complexity	Point-like^26,60,64^/Linear^69^/Spectral FLIM^65^/FLIM-FRET^68^	Increased sensitivity (especially in the red and NIR regions), shared resources such as TDCs, improved timing resolution
FLIM - Widefield^10,56,59^	ICCD, MCP	2D	+ Video rate lifetime estimation (on-chip and/or on FPGA), compact all-solid-state gating, noiseless read-out − Fill factor, non-uniformity, large data rate, dynamic range limited by the TDC conversion rate (TCSPC) or counter bit depth (gated)	MegaFrame^51,86^/SwissSPAD2^73^/Analogue timing^37,38^	Increased sensitivity (especially in the red and NIR regions and fill factor), spatial resolution (smaller pixels) and uniformity, dedicated lifetime estimation on-chip, multi-bit counters
FLIM - Multibeam	n/a	2D	+ Increased count rate due to pixel parallelisation, real-time lifetime estimation on an FPGA − Sensor alignment	MegaFrame^91,92^/Vitali^93^	Optimised optical alignment setup
FCS - Multibeam^116^	n/a	2D	+ Increased count rate due to pixel parallelisation − Sensor alignment, DCR, afterpulsing	Vitali^93^/Kloster-Landsberg^105^	Optimised optical alignment setup
Widefield FCS^100,116,180^	EMCCD, sCMOS	2D	+ Frame rate, noiseless read-out − Fill factor, sensitivity, dynamic range limited by 1-bit counters, afterpulsing	RadHard2^106^/SwissSPAD^110^	Multi-bit counters, on-chip/on-FPGA autocorrelation and cross-correlation calculation
Single-molecule - Multibeam^112,116^	APDs	[Custom SPADs] Linear, 2D (small)	[Custom SPADs] + Increased count rate due to pixel parallelisation − DCR, non-uniformity, non-integrated electronics	Ingargiola^120,121^	[Custom SPADs] Increased sensitivity (in the red region), reduced DCR, improved non-uniformity, 3D integration with CMOS read-out chip
SRM^123,124^	EMCCD, sCMOS	2D	+ High-speed, noiseless read-out (→ analysis of μs blinking, precise estimation of the blink duration) − DCR non-uniformity, sensitivity	SwissSPAD^129^/Dutton^130^	Increased sensitivity, decreased DCR non-uniformity and percentage of “hot” pixels by SPAD miniaturisation
Time-resolved Raman^134,135,138^	(I)CCD	Linear	+ Fluorescence background rejection by means of on-chip time-gating and/or time-stamping, compact systems − Sensitivity, spatial resolution vs. gate length/uniformity	Maruyama^139,140^, Nissinen^143^, Rojalin^144^, Krstajić^67^	Increased sensitivity (especially in the red and NIR regions), reduced gate length, increased time-gating uniformity, pixel miniaturisation
NIROT^149,150^	PMT, SiPM	2D	+ Increased count rate due to pixel parallelisation and on-chip time-stamping electronics − Sensitivity, data rate, dynamic range	Piccolo^95,154^	Increased sensitivity (especially in the red and NIR regions) and dynamic range (e.g. through gating), on-chip data compression
Q-LSRM	n/a	2D	+ On-chip timing correlations − Sensitivity, cross-correlations	SPADnet^163^, Gasparini^36^	Crosstalk minimisation
PET^47^	PMT	SiPM	+ B-field insensitivity, timing resolution, on-chip time-of-arrival measurement (digital SiPM) − Sensitivity, DCR, data rate (multi-digital approach)	Carimatto^23^, SPADnet1^49^	Increased sensitivity and timing resolution, data compression

*APD* avalanche photodiode, *EMCCD* electron-multiplying charge-coupled device, *hybrid* hybrid photomultiplier, *ICCD* intensified charge-coupled device, *MCP* microchannel plate, *PMT* photomultiplier tube, *sCMOS* scientific CMOS, *custom SPADs* non-standard CMOS SPADs

## Fluorescence lifetime imaging

Fluorescence lifetime imaging (FLIM) is a non-invasive measurement technique for applications where the fluorescence intensity does not provide sufficient information or discrimination. Common usage of FLIM is found in the study of living tissues and cells at the molecular level, because the fluorescence lifetime is insensitive to fluorescence intensity and to the corresponding probe concentration, at least to a reasonable extent^56^; however, the samples should not be subject to excessive illumination intensities, to avoid phototoxicity and photobleaching. Other advantages are the detection of lifetimes that can be dependent on pH, temperature, oxygen concentration and viscosity levels, thereby enabling the detection of effects that cannot be observed with simple fluorescence intensity measurements.

The slow acquisition speed (<10 Hz) is the main limitation of standard FLIM setups. While the photophysics at the molecular level contributes to this, the detection system can also impose major speed limitations. Time-correlated single-photon counting (TCSPC), which requires timestamps of individual photons, is often the detection method of choice due to its very high precision, but the underlying hardware and data acquisition are hard to scale to large multichannel arrays; scanning is, therefore, required when TSCPC is used in an imaging setup. Time-gated sensors have also been employed, including large sensitive areas; they rely on one or more (moving) gates to recover the timing information, and thus the lifetime, at the expense of a reduction in the overall sensitivity, as discussed in the “Architectures” section.

Interested readers can refer to refs. ^9,10,57–59^ for the background literature on FLIM and the related sensors, techniques and applications. In the following subsections, we will focus on standard CMOS SPAD implementations for FLIM and how these implementations have been engineered and employed to address the aforementioned limitations.

### Point-like FLIM

Point-like FLIM systems offer increased signal-to-noise ratios by combining the individual outputs of several pixels. An example of such an approach is represented by the FluoCam^26^ system, which comprises a 60 × 48 SPAD array^31^, with two 8-bit time-gated counters in each pixel. The gates can be externally programmed to shift in steps of ~12 ps, to cover a full laser repetition period with high accuracy. The two counters, therefore, allow a precise reconstruction of the fluorescence response, even when significant photobleaching distorts the signal, including sub-nanosecond lifetimes. The integration times are on the order of several minutes, but can be substantially reduced by resorting to more recent designs and/or technology nodes.

**Fig. 3:**
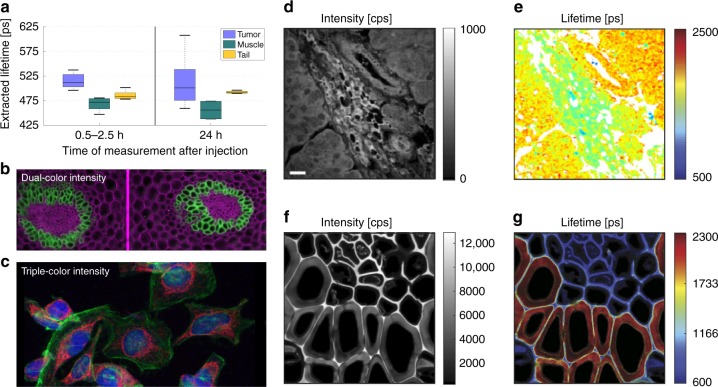
Example fluorescence intensity and/or lifetime results. **a** FluoCam system used in a point-like mode for the study of monomeric ICG-c(RGDfK) injected in a mouse with a glioblastoma mouse model. A subtle lifetime shift between tumour and non-tumour tissue is observed^26^. **b** Dual-colour intensity fluorescence image of a thin slice of a plant root stained with a mixture of Safranin and Fast Green, taken with the SwissSPAD widefield time-domain gated array^178^. **c** Triple-colour intensity fluorescence image of HeLa cells labelled with DAPI, Alexa 488 and Alexa 555, taken with SwissSPAD2^73^. **d**, **e** Label-free FLIM of an unstained liver tissue excised from a tumourigenic murine model^65^, imaged with a 64 × 4 SPAD array^18^. **f**, **g** A Convallaria FLIM measurement performed with a linear 32 × 1 SPAD array^70^. The images are reprinted from refs. ^26,65,70,73,178^

The FluoCam system has been used in several in vivo studies to demonstrate the capabilities of such an approach, employing indocyanine green (ICG)-modified derivatives, such as ICG-RGD, which target the *α*_v_*β*_3_ integrin; the final goal was to explore the feasibility of surgical applications with exogenous NIR targeted fluorophores^26,60^. The system was capable of discriminating between healthy (muscle and tail) and cancerous tissues in a mouse with a glioblastoma mouse model, even though the lifetime difference was only ~50 ps (10% level in this case) between the lifetimes of the bound and unbound fluorophores as shown in Fig. 3a.

In TCSPC mode, the detection system’s limitations introduce pile-up effects for detection rates >0.1 photon/laser cycle, causing an underestimation of the lifetime. Pile-up correction using standard SPAD detectors has been discussed in detail by Léonard et al.^61^, who show that low count rates are not necessarily needed to avoid pile-up, at the expense of an increase in the lifetime estimation variation. In integrated SPAD detectors, multiple subsystems contribute to pile-up. A detailed analysis of the influence of the SPAD, timing and routing dead time on the lifetime estimation can be found in ref. ^62^, with experimental results based on a mini-silicon photomultiplier (32 × 32 SPAD pixels) implemented in a 130 nm CMOS process, and featuring an on-chip lifetime estimator^63,64^. The outputs of all pixels are routed towards 16 TDCs that can time-stamp up to 8 photons per excitation period. These timestamps are then processed in a centre-of-mass module to derive the fluorescence lifetime. This technique has been validated with reference samples with relatively long lifetimes of over 1 ns, demonstrating that reliable lifetimes can be estimated, with a proper architecture, at photon count rates that go well beyond the classical pile-up limit.

### Linear SPAD arrays and corresponding FLIM applications

The advantages and disadvantages of linear architectures have already been discussed at the beginning of the Array architecture section. Pancheri and Stoppa^18^ implemented a 64 × 4 linear SPAD array (overall size of 1660 × 104 μm^2^ in 0.35 μm CMOS technology) targeted for FLIM. The four SPADs in each column were connected to the same read-out channel, creating macro-pixels to reduce the influence of the single SPAD dead time (~50 ns). This increased the photon throughput of a 15.8 × 63.2 μm^2^ macro-pixel. The chip also featured four time-domain gates that were connected to four separated counters, enabling the construction of on-chip histograms of the photon-arrival times with four bins and data compression. This sensor was later used^65^ for spectrally resolved FLIM (sFLIM or *λ*FLIM), a setup that enables the separation of molecules by both the fluorescence emission wavelength and the fluorescence lifetime^66^. An example of a corresponding tissue image is shown in Fig. 3d, e. The *λ*FLIM system simplifies the discrimination of different fluorophores and enables the simultaneous study of donor and acceptor molecules.

More recently, Krstajić et al. presented a linear 256 × 2 SPAD array with a pixel pitch of 23.7 μm and a high fill factor of 43.7%, implemented in a 130 nm CMOS process^24,67,68^. Each pixel was connected to one TDC with a 40 ps LSB. The sensor also featured an on-chip centre-of-mass (CMM) calculation for mono-exponential fluorescence lifetimes, enabling to output lifetimes at a 200 Hz line rate with up to 65 kphotons/pixel (limited by the SPAD dead time). Alternatively, the chip can output per-pixel TCSPC histograms with a 320 ps bin resolution. Multicolour microspheres and skin autofluorescence lifetimes were measured, with a data acquisition time of 5 min for TCSPC data, to be compared with 2 or 200 ms when operating in CMM mode for fluorophores in cuvette or skin autofluorescence, respectively. This chip is currently being used in the (https://www.proteus.ac.uk/) EPSRC UK PROTEUS project, targeting in vivo, in situ microendoscopic instrumentation for the diagnosis of lung diseases. Erdogan et al.^69^ designed a next generation linear array, in the same 130 nm CMOS process, extending the resolution to 1024 × 8. The chip featured 512 TDCs and on-chip histogram generation that decreases the output data rate and mitigates the I/O and USB bottlenecks.

Linear arrays can also be used as single-point detectors, for example by means of optical 1D to 2D transformations, to reduce the effect of single SPAD dead time and increase the throughput in FLIM measurements^70^; an example of the corresponding results is shown in Fig. 3f, g. This and other approaches could pave the way for high-throughput biotechnological applications, such as high-throughput screening or cell sorting^61,71^, based on nanosecond-lived fluorophores.

### Widefield time-domain gated FLIM

Gated SPAD arrays are in principle easier to implement over large areas than TCSPC solutions, and thus provide an appealing path to all-solid-state widefield, time-resolved imaging.

An initial implementation of a time-gated 128 × 128 SPAD array with 1-bit memory combined an on-chip 600 ps delay line and an off-chip 200 ps delay line for gate shifting^20^. DNA molecules labelled with Cy5 were placed directly on the chip surface and the lifetime was measured. Time-gating enabled an excitation elimination without the need for dichroic mirrors.

SwissSPAD^15^ is a 512 × 128 SPAD array with an in-pixel 1-bit memory and time-gating capability. The 1-bit frames are read out at 156 kfps, while time-gating enables independent global exposures as short as 4 ns. The gate position can be shifted in 20 ps steps with respect to a reference signal. This enables the reconstruction of the exponential lifetimes per pixel. The implemented pixel, which contains 12 transistors, has a 5% fill factor due to the 0.35 μm manufacturing process. With the use of microlenses, the effective fill factor is increased to 50–60% for collimated light, as often featured in microscope output ports^44^; a representative fluorescence intensity image is shown in Fig. 3b.

Early characterisation of SwissSPAD (without microlenses) for FLIM measurements was performed with reference data sets^72^; the sensor could properly extract the lifetime of fluorophores in the nanosecond range. Ulku then designed SwissSPAD’s successor, SwissSPAD2^29,73,74^, a 512 × 512 SPAD array—the largest time-resolved SPAD image sensor to date—with a higher PDP and a lower DCR, based on a similar architecture. A triple-colour fluorescence intensity image is shown in Fig. 3c. Further research on widefield time-domain gated FLIM with microlense-enabled versions of SwissSPAD architectures is ongoing, including real-time phasor-based measurements^73,75^.

Following up on the proof-of-concept work by Pancheri et al.^76–78^, Perenzoni et al.^38^ designed a 160 × 120 SPAD imager with gating, but with multi-bit memory. The gate can be set as short as 750 ps, with rise and fall times down to 200 ps, and a frequency of 50 MHz. Instead of a 1-bit memory, this chip uses an analogue counter, enabling multiple photon accumulations per frame at the cost of introducing ADCs. The pixel pitch is 15 μm, resulting in a 21% fill factor in 0.35 μm high-voltage CMOS technology. Gyongy et al.^30^ pursued an *n*-well shared pixel approach to achieve a high native fill factor of 61% for a 256 × 256 SPAD array with 4 ns gates, 600 ps fall times with an on-chip delay generator, and a pixel pitch of 16 μm.

### Widefield TCSPC FLIM

A 64 × 64 40 μm pitch pixel array designed in 0.35 μm high-voltage CMOS technology, featuring 64 column-parallel TDCs and a timing resolution of ~350 ps, represented an early implementation of a SPAD TCSPC array work^79,80^. However, the maximum PDE of 0.1% was relatively low.

The MEGAFRAME32 high-performance sensor was smaller (32 × 32 SPADs) but adopted a radically different architecture, based on 50 ps, 10-bit in-pixel TDCs, working at a maximum rate of 500 kfps^21,81^, and recording either time-correlated data (one time-stamp per pixel), or time-uncorrelated data (6-bit counting). In the former operation mode, up to 0.5 billion timestamps could be generated per second^82^. The fill factor (1%) was adversely affected by the large in-pixel electronics; on the positive side, this demonstration stimulated pioneering microlens research to bring the fill factor back up. MEGAFRAME32 was extensively employed to explore bio-applications and subsequently extended to a 160 × 128 array (MEGAFRAME128), adding peripheral intelligence (data compression and CMM pre-processing)^33,83,84^.

Gersbach et al. reported early high frame rate FLIM proof-of-concept investigations^21,85^ with MEGAFRAME32. Li et al.^50,51,86^ illustrated how firmware-based rapid lifetime estimation algorithms, such as CMM (centre-of-mass method), make full use of the large number of available timestamps to enable video-rate (50 fps) real-time FLIM operation. An example of the corresponding in vivo two photon FLIM data, with both the intensity and the lifetime, is shown in ref. ^86^ using an FITC-albumin probe, which was injected into a rat bearing a P22 tumour and measured 100 min after the injection. A clear distinction between the blood vessels and the tumour tissue could be observed in the lifetime image (bi-exponential decay), in contrast to the intensity image. Another widefield FLIM application of the same sensor, coupled to DNA microarrays, was reported in ref. ^87^ employing a TIRF excitation geometry. Distinct lifetime signatures, corresponding to dye-labelled HCV and quantum-dot-labelled HCMV nucleic acid targets, could be distinguished over 320 pixels, with concentrations as low as 10 nM and an exposure time of 26 s.

A different architecture was selected by Field et al. for their 64 × 64 array, in a standard 0.13 μm CMOS process, and reported in ref. ^34,35^, namely with one TDC per pixel (LSB of 62.5 ps) placed at the column level; this led to a pixel pitch of 48 μm. The sensor was aimed at video-rate operation (100 fps), but the corresponding extreme data rate of 42 Gbps led to a high power consumption (14.5 mW/pixel).

Parmesan et al.^37^ have chosen to emphasize small pixel pitches (8 μm), while still maintaining a pixel fill factor of nearly 20%, by resorting to an architecture based on in-pixel time-to-amplitude (TAC) converters, with a global ramp voltage. This enabled the design of a large 256 × 256 array, which could work either interfaced to external TDCs (with optimal timing performance, but resulting in a slower system) or using an on-chip coarse flash ADC (with a lower temporal resolution).

### Multibeam FLIM

Multibeam architectures enable increased photon throughput and reduced FLIM acquisition times. Coelho et al. and Poland et al. used MEGAFRAME32 with a spatial light modulator (SLM) for multibeam multiphoton FLIM, increasing the throughput by the number of parallel beamlets^88–91^. The fill factor does not decrease the sensitivity in such setups, because beams are concentrated onto the active area of the SPADs. FLIM data of live cells (MCF-7 human carcinoma cells) labelled with green fluorescent protein were acquired within 500 ms, albeit at a reduced accuracy. This approach was extended in ref. ^92^ with a new CMM method, mostly implemented in hardware, which was capable of pixel level background subtraction and did not require prior knowledge of the expected lifetime; real-time operation was obtained, with a reduced accuracy compared to the accuracy of TCSPC mean squared fitting techniques.

Vitali et al.^93^ also used a multibeam approach with a 32 × 32 array (square pixels of 100 μm with a 20 μm circular SPAD and 8-bit counters) and performed FLIM of eGFP in living HEK293-T cells. The SPAD sensor was implemented in a standard CMOS process and included time-gating with a minimum gate width of 1.5 ns and delay steps below 100 ps.

### FLIM-FRET

FRET uses interactions between two different chromophores (light-sensitive molecules) that non-radiatively transfer energy from a donor to an acceptor molecule. The energy is transferred only when the distance between the two molecules is small enough (nm scale, establishing long-range dipole–dipole coupling) and when there is sufficient overlap between the emission spectrum of the donor and the excitation spectrum of the acceptor. During this coupling, one can observe a decrease in the donor fluorescence and an increase in the acceptor fluorescence. A typical application is the study of protein–protein interactions and the measurement of distances between molecular groups in protein conformations^94^.

FLIM-FRET not only measures the fluorescence intensity change in the donor and acceptor emissions, but also the shortened lifetime of the donor molecule as a result of quenching^56^. By measuring the ratio between the quenched and non-quenched lifetime, the donor–acceptor interactions can be quantified independently from the molecule concentrations within a diverse sample (in contrast to emission intensity FRET).

Poland et al. implemented a MEGAFRAME32-based multifocal FLIM-FRET detector system combined with the optical setup mentioned in the Multibeam FLIM section^88–91^. While scanning protein–protein interactions in live cells with a frame time of 500 ms, they studied changes in FRET interactions between epidermal growth factor receptors (EGFR) and adapter proteins Grb2, as well as a ligand-dependent association of HER2-HER3 receptor tyrosine kinases. Kufcsák et al.^68^ used 5-carboxyfluorescein as a donor and methyl red as an acceptor in FRET for thrombin detection. Thrombin cleaves the connection between the donor and acceptor, separating them in space and removing the energy transfer.

### Conclusions

The applicability of SPAD arrays for FLIM was limited in the early implementations by the relatively low PDP and fill factor (multibeam FLIM being an exception to the latter, due to the corresponding peculiar optical setup), combined with high a DCR. While increasing the sensitivity in the red and NIR regions is still important on the SPAD development roadmap, substantial progress has been made in all performance metrics, as also summarised in the overall “Conclusions” section and in Table 3.

SPAD arrays in standard CMOS technology have a clear advantage when parallelising data acquisition is important (including in widefield setups) and/or when real-time operation, e.g. a lifetime calculation, is needed. In general, it is more difficult to operate SPAD arrays than (scanning) single-point detectors, which in addition can be optimised for a maximum sensitivity and/or temporal resolution.

SPAD arrays with resource parallelisation can be used as single-point detectors to reduce the effect of dead time and increase the throughput in FLIM measurements. On-chip histogram generation and lifetime estimation further reduce the data rate. This could pave the way to high-throughput biotechnological applications, such as high-throughput screening or cell sorting^61,71^, based on nanosecond-lived fluorophores.

1D and 2D arrays can also eliminate scanning in one and two dimensions, respectively. In this case, an increase in the throughput compared to that of scanning systems is obtained if the non-scanning system dead time T1 is shorter than the array resolution R multiplied by the scanning system dead time T2, i.e. T1 < R × T2. This approach enabled, for example, video-rate imaging lifetime estimation^51^.

Compact, all-solid-state time-domain gated implementations have emerged at the forefront of high-spatial resolution widefield FLIM. Ongoing work is expected to further improve the gate timing precision^73^, whereas an additional increase in the imaging speed will likely occur by implementing multi-bit per-pixel counters. Recent 2D arrays targeting widefield TCSPC FLIM have increasingly shared TDC resources to combine a higher photon throughput with reduced area and power consumption^46,95,96^.

On the application side, the precision of the lifetime estimation depends on the number of detected photons and the photon efficiency of the used instrumentation and method, which can be characterised by the *F* value ($F = \sqrt N \frac{{\sigma _\tau }}{\tau }$, where *N* is the number of detected photons, *τ* is the lifetime and *σ*_*τ*_ the lifetime estimation precision)^97^. “Ideal” TCSPC systems are widely assumed to have *F* = 1, whereas TCSPC SPAD arrays have reported *F* values up to 1.5 (refs. ^51,98^), and the most recent time-domain gated implementations have estimated *F* values between 2 and 5 (refs. ^74,98,99^). If, for example, a 5% lifetime estimation precision is required (e.g. 100 ps for a 2 ns lifetime), such as for demanding applications like FLIM-FRET, one can estimate the required number of detected photons with $N = \frac{{F^2}}{{0.0025}}$. Therefore, a system with an *F* value of 2 would need each pixel to acquire 4× more photons than an ideal system to achieve an equivalent lifetime estimation precision, e.g. 1600 photons instead of 400 to reach a 5% level.

Therefore, higher *F* values increase the constraints for demanding live-cell imaging, which imposes limitations on both the excitation intensity (due to photobleaching) and excitation duration (due to movement). Reaching a reasonable frame rate, e.g. 10 fps, with a gated widefield array calls for a maximum count rate capability of ~160 kcps per pixel ($\frac{{{\mathrm{1600}}\,{\mathrm{detected}}\,{\mathrm{photons}}}}{{100\,{\mathrm{ms}}}} \times 10$ to avoid read-out pile-up when working with binary frames) to meet the previously mentioned 5% precision level. Such a requirement is in the range of what is achievable with recent time-domain gated arrays^15,30,73,74^. A similar conclusion can be reached for TCSPC SPAD arrays, such as refs. ^46,51,95^, whereas larger implementations call for on-chip intelligence, such as histogram generation^96,98^, to relax the constraints on the read-out bandwidth. Note that these estimations assume that the application setup delivers a sufficient photon flux to the sensor, and that the latter features a PDE high enough to reach the requested number of “detected photons” per image frame.

## Fluorescence correlation spectroscopy

Fluorescence correlation spectroscopy (FCS) measures fluorescence intensity fluctuations in time, with the aim of estimating the concentration and diffusion coefficients of fluorophores, including in live cells. These parameters are extracted from the autocorrelation of the temporal intensity fluctuations. Faster sensors and imagers enable the study of smaller and faster molecules^100^. In a widefield setup, the correlation between signals from distant volumes measures the direction and velocity of the flow between the volumes under investigation.

### Multibeam FCS

The multibeam parallelisation principle introduced in the Multibeam FLIM section can also be applied to FCS setups, generating a large number of laser foci using SLMs or diffractive optical elements (DOEs), while taking care to minimise the background signal generated by out-of-focus light. A single SPAD or a group of SPADs are then used to detect fluorescence from each laser focus. Goesch et al. used a small, fully integrated 2 × 2 CMOS SPAD array in the pioneering work on multibeam FCS reported in ref. ^101^. The multibeam concept was later extended to 8 × 8 spots to image bright 100 nm diameter fluorescent beads in solutions using a 32 × 32 SPAD array^102^. The latter was then employed, together with the 32 × 32 multibeam setup previously described in ref. ^93^ to perform FCS of quantum-dot diffusion in solution^93,103,104^. The researchers used a DOE to generate 32 × 32 spots with a pitch of 100 μm and a diameter of 12.5 μm in the image plane (to match the sensor dimensions).

Independently, Kloster-Landsberg et al.^105^ used the 32 × 32 MEGAFRAME32 sensor to perform multifocal FCS with live cells, employing a frame time of 2 μs. In this setup, 3 × 3 laser foci were used for experiments with free eGFP in HeLa cells. A larger multibeam array could not be employed due to the high crosstalk between closely packed spots that emerge in this kind of setup.

### Widefield SPIM-FCS

FCS coupled with single plane illumination microscopy (SPIM-FCS) enables a faster characterisation of 3D samples, and records intensity fluctuations over a widefield plane. By illuminating a micrometre-thick light sheet in the *z*-section, the out-of-focus light, photobleaching and photodamage can be minimised. The first SPIM-FCS data obtained with a SPAD array were presented in ref. ^106^. The work compared the RadHard2 32 × 32 SPAD array^107^ with EMCCD and sCMOS cameras. The RadHard2 camera achieved very high read-out speeds, up to 300 kfps, enabling the extraction of diffusion coefficients down to 3 μs with a better precision than the EMCCD and sCMOS cameras. The camera was coupled with a real-time 32 × 32 autocorrelator, implemented on an FPGA^53^, which allowed real-time autocorrelation calculation for small molecules in solution, with diffusion coefficients down to 10 μs. However, RadHard2 did not feature microlenses, which obviously affected the overall sensitivity and limited its in vivo applicability.

**Fig. 4:**
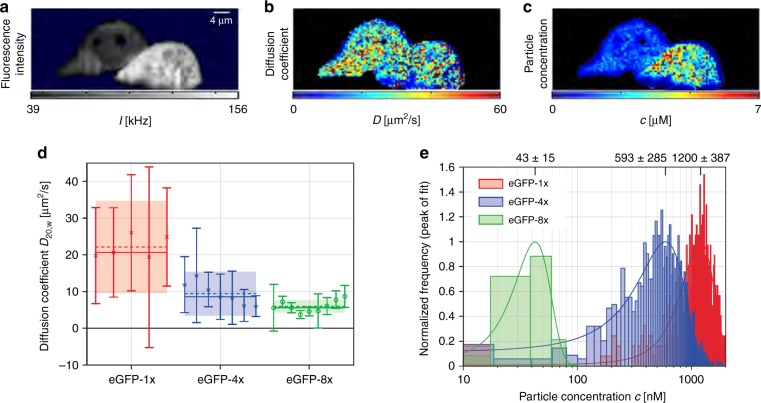
Widefield SPIM-FCS images of monomeric eGFP oligomers in HeLa cells as recorded with a SwissSPAD widefield imager. **a** Fluorescence intensity, **b** diffusion coefficient and **c** dye concentration. **d** Diffusion coefficients for three HeLa cells expressing different oligomers. **e** Particle concentration for the three HeLa cells with different oligomers. The images are reprinted from ref. ^110^

Widefield in vivo SPIM-FCS with SPAD arrays was first demonstrated with a microlensed version of SwissSPAD^15,44,72^ by Buchholz et al. and Krieger et al.^108–110^. The FCS results in HeLa cells are shown in Fig. 4 for three different oligomers of eGFP. The autocorrelation curves of these measurements featured afterpulsing-like increased correlations at short time lags; these artefacts were mitigated by using spatial cross-correlations^111^, which also allowed the determination of absolute diffusion coefficients without a prior calibration. Although the sensitivity needs to be further increased, this work showed that SPAD arrays can measure protein diffusion in live cells with a better SNR than EMCCD cameras and a minimum lag time of 10^−5^ s. The correlation algorithms were also extended to GPUs.

### Conclusions

The early works demonstrated how multibeam FCS can overcome the limitation of a low native fill factor by concentrating laser foci on the active area of the SPADs. The main challenge in such systems is the optical alignment. Widefield SPIM-FCS has benefited from the high frame rate and noiseless read-out of large binary SPAD arrays, leading to a minimum lag time of 10 μs. Although microlenses were demonstrated to be effective, a further increase in the PDP is needed to improve the detector’s sensitivity, particularly for in vivo measurements. Additional CMOS-enabled functionalities, such as on-chip/on-FPGA autocorrelation functions, might assist in reducing the data rate. Finally, low afterpulsing is also a key requirement for FCS; if necessary, the spatial resolution can be reduced by employing cross-correlation of 2 × 1 or 2 × 2 pixels to eliminate the effect of afterpulsing.

## Single-molecule techniques

Single-molecule fluorescence spectroscopy exploits a low concentration regime to excite individual molecules in a very small volume, and collect rare, burst-like fluorescence emission events corresponding to the transit of individual molecules (whereas in FCS the concentration is such that ~1 or more molecules are present in the excitation volume at any time—see ref. ^112^ for a thorough, SPAD-oriented analysis). The number of generated photons is small (a few dozen or less in a large fraction of bursts) and, therefore, the detection is quite challenging. This in turn imposes stringent requirements on the photosensor(s), in terms of sensitivity (including in the red region, where standard CMOS is not advantageous due to the low absorption of silicon), fast response time, low noise (read-out or DCR for SPADs) and high count rates, in order to separate successive or nearby molecules. The measurement times are usually long to accumulate sufficient statistics.

Several (small) arrays of devices fabricated in custom technologies have been employed over the years with success^2,112–117^, and parallelisation strategies have again been adopted to increase the overall throughput, often employing sophisticated optical setups (multispot excitation and detection). For example, a parallel 8-spot single-molecule FRET (smFRET) analysis system was described in refs. ^118,119^ using 8-pixel SPAD arrays and in ref. ^120^, where the authors tackled real-time kinetic analysis of the promoter escape by bacterial RNA polymerase, confirming results obtained by a more indirect route.

An extension to 48 excitation spots and two 48-pixel SPAD arrays was detailed in ref. ^121^, employing two excitation lasers to separate species with one or two active fluorophores. Apart from successfully tackling the multispot setup issues, the resulting smFRET capabilities were shown for a set of doubly labelled double-stranded DNA oligonucleotides with different distances between the donor and acceptor dyes along the DNA duplex. The resulting acquisition times were drastically reduced to seconds. This could in turn enable high-throughput screening applications and real-time kinetics studies of enzymatic reactions, potentially propelling single-molecule analysis from a niche technology to a mainstream diagnostic and screening tool^120^.

For a comprehensive overview of this application, where (small) arrays of SPAD devices fabricated in custom technologies dominate, we refer the interested readers to Table 3.

## Localisation-based super-resolution microscopy

The optical resolution is fundamentally limited by diffraction, whereby Abbe defined the corresponding limit to be $\frac{\lambda }{{2n{\mathrm{sin}}\theta }}$ , where *λ* is the emission wavelength, *n* is the refractive index and *θ* is the half-angle of the cone of light that enters into the objective^122^. Several super-resolution techniques have emerged over the years to overcome the diffraction limit, enabling resolution enhancements from initial values of 200 nm down to 10 nm^123^. One such technique uses sparsely activated (blinking) fluorescent molecules for single-molecule localisation^124^, whereby the location of each molecule is determined by the centre of its point-spread function. The final pointillistic image is then reconstructed by merging thousands of frames with hundreds of thousands of localisations, with a resolution limited by the localisation precision and labelling density. Owing to the long frame sequences, durations of tens of seconds to minutes are necessary to reconstruct the final super-resolved image; this time could be shortened by employing higher frame rates and/or stronger laser intensities.

**Fig. 5:**
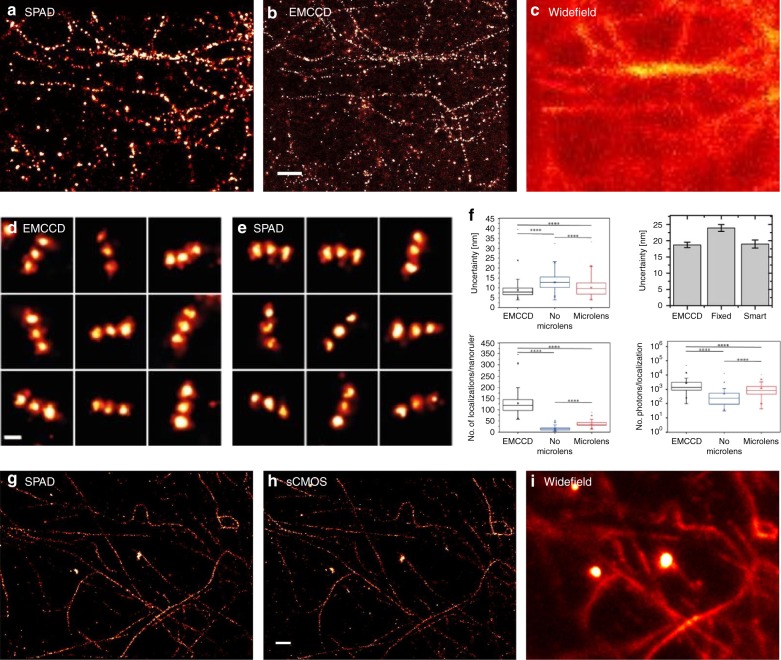
SPAD super-resolution images. **a** The first super-resolution image captured with SwissSPAD, compared to **b** EMCCD and **c** widefield images. The images show the microtubuli of an U2OS cell labelled with Alexa Fluor 647, in Vectashield^129^. **d**, **e** Comparison of the SPCImager using “smart” aggregation and microlenses with an EMCCD. The images show multiple GATTA-PAINT 40G nanoruler localisations^45^. **f** Comparison of the differences in localisation uncertainty with and without “smart” aggregation and the impact of the microlenses^45,130^. **g** SwissSPAD super-resolution image of microtubuli labelled with Alexa 647 in OxEA buffer compared to **h** sCMOS and **i** widefield images^129^. The white bar indicates 1 μm. The images are reprinted from refs. ^45,129,130^

Antolovic et al.^125^ demonstrated the first localisation-based super-resolution (SRM) images obtained with SPAD arrays, which are compared in Fig. 5a to EMCCD (Fig. 5b) and widefield (Fig. 5c), by employing the SwissSPAD imager^15^. Microlenses were deposited on the SPAD array to improve the fill factor from the native 5 to 60%^44^, which was a key enabler for applications in which the sensitivity is critical. The image resolution was analysed with the Fourier ring correlation method^126^, yielding a resolution of ~100 nm. The estimated localisation uncertainty^127^ was 30 nm, with 200 photons per localisation, compared to 15 nm obtained with an EMCCD camera, using 1800 photons per localisation. In other terms, although almost 10× more photons were acquired with the EMCCD camera, the localisation results were only 2× better; the reason was that the SNR, which should indeed increase by $\sqrt {10}$, was reduced by $\sqrt 2$ due to the multiplication noise in the EMCCD camera itself, which arises from the electron multiplication process. Later results with an improved buffer^128^, shown in Fig. 5g, h, yielded 800 photons per localisation with a localisation uncertainty of 10 nm for an sCMOS camera, while SwissSPAD collected 100 photons under the same conditions, leading to an uncertainty of 20 nm^129^.

When compared to standard EMCCD and sCMOS cameras, SPAD imagers eliminate the read-out noise by utilising a direct photon-to-digital conversion—the digital nature of SPAD imagers enables fast and noiseless read-out. These properties of SPAD imagers were used in three ways. First, Gyongy et al.^130^ used temporal oversampling and “smart” aggregation to determine the start and end of fluorophore blinking in a more accurate manner to minimise background noise. Second, Antolovic et al.^129^ investigated the performance of the localisation algorithm for different frame rates. Finally, SPAD imagers were used to perform a widefield analysis of fluorophore blinking in the μs range^129^.

In terms of the first point, Gyongy et al. used the 320 × 240 SPAD SPCImager^25^ for localisation-based super-resolution imaging^45,130^. By employing microlenses, they improved the effective fill factor from 27 to 50% and used this sensitivity enhancement to demonstrate a resolution of 40 nm. A GATTA-PAINT 40 G nanoruler was used as a reference, and the corresponding results are shown in Fig. 5d–f. “Smart” aggregation decreased the localisation uncertainty by 20%, and the simulations showed a potential improvement by 50%. Although the SPAD results have a localisation uncertainty comparable to that of EMCCDs, a 3× lower sensitivity in the green leads to a lower number of localisations.

Concerning the second point, Antolovic quantified the optimum frame rate given the exponential distribution of fluorophore blinking^129^, if oversampling and smart aggregation could not be used. Oversampling leads to a higher localisation uncertainty, while undersampling causes a decrease in the number of localisations.

Finally, SwissSPAD’s high frame rate of 156 kfps was used to explore additional high-frequency μs blinking, which in general reduces the total number of collected photons and thus the final resolution. As an example, the percentage of Alexa 647 molecules blinking at a high frequency decreased from >68 to <30% when switching from an MEA to an OxEA buffer^129^. This indicated that high-speed SPAD imagers could be beneficial for fluorophore/buffer design and optimisation.

### Conclusions

As the previous discussion has indicated, the fundamental differences between EMCCD, sCMOS and SPAD imagers do also need to take into account the different noise contributions and achievable frame rates, in addition to the overall sensitivity. A theoretical comparison between these imagers, when applied to localisation-based super-resolution microscopy, has been conducted by Krishnaswami et al.^131^. EMCCDs feature a quantum efficiency of 90%, but also exhibit multiplication noise that effectively reduces this value by half^132^; the noise performance of EMCCDs is very uniform due to the serial read-out, but the achievable frame rate is low. The sCMOS cameras parallelise the read-out and increase the frame rate at the expense of mismatches in the analogue electronics. SPAD imagers eliminate read-out noise with fully parallel digitisation and can thus achieve very high frame rates for binary (1-bit) frames. If multiple 1-bit frames are added to achieve an 8- to 12-bit depth, which might be necessary or not depending on the application, their frame rates become comparable to those of the sCMOS cameras. On-chip counters will eliminate this bottleneck, enabling high frame rates with a higher bit depth. In addition, the high speed and picosecond temporal resolution of TDCs connected to SPADs can open up new avenues, such as video-rate localisation super-resolution and multicolour imaging. The SPAD digitisation also limits the noise contributions solely to the DCR (and afterpulsing), and although DCR levels are acceptable for super-resolution applications, the percentage of “hot” pixels needs to be further reduced.

## Raman spectroscopy

Raman spectroscopy provides data on the chemical composition and molecular structure of a compound in a non-destructive and label-free manner^133–135^, with applications to both in vitro and in vivo tissue diagnostics. This “fingerprinting” technique relies on inelastic light scattering with vibrating molecules, whereby Stokes-scattered Raman photons are redshifted. Raman spectroscopy has seen a surge in biophotonics applications in the last couple of decades^134^, although it typically suffers from a weak scattering cross-section, leading to long overall acquisition times (unless used, for example, in combination with other sampling techniques that reduce the area to be interrogated, line scanning, or multifocal/widefield setups). As such, the Raman signal is often overshadowed by the sample (auto)fluorescence itself; when working with biological samples, it might, therefore, be of interest to move towards the NIR range, where autofluorescence is weaker, although the resulting SNR needs to be weighted by the corresponding reduction in the scattered intensity, which decreases with the 4th power of the excitation wavelength. The use of coherent techniques such as non-linear anti-Stokes Raman scattering (CARS), resulting in blueshifted radiation, can enhance the scattered signal and circumvent these limitations, enabling, for example, rapid chemical imaging, at the expense of appropriate tunable, femtosecond laser sources^134^. Penetration into deep tissues, which is of particular relevance for in vivo medical diagnosis or when looking at live cells growing in 3D cultures, can be enhanced with recently developed methods such as spatially offset Raman spectroscopy (SORS) and transmission Raman spectroscopy (TRS); interested readers can consult a recent review^136^.

A disadvantage of moving to the NIR region to reduce the (auto)fluorescence background is that the sensitivity of standard CCD/CMOS imagers decreases, and this is particularly true for SPAD imagers developed with standard CMOS technology, i.e. the primary focus of this review. However, SPADs allow the design of compact, all solid-state detectors for Raman spectroscopy operating in time-resolved mode, whether via very short gates, ideally in the 10–100 ps range given the nature of the Raman signal (very fast emission compared to the fluorescence background, which is typically in the ns range)^137,138^, or based on time-stamping, e.g. with TDCs; this mode of operation also reduces the DCR contribution and thus enhances the overall SNR. Several linear SPAD-based systems have indeed been recently developed, usually comprising one or a few lines, targeting initially applications such as mineralogy and subsequently biophotonics.

One of the largest reported SPAD arrays is a 1024 × 8 system^139–141^, which was applied with success to mineral samples, where the background fluorescence timescale is longer than for typical biological samples of interest to us. The array featured 1-bit counters and a global gate as short as 750 ps (with a corresponding standard deviation of 120 ps), which could be shifted in 250 ps steps. The use of a standard 0.35 μm CMOS process led to a maximum PDE of 9.3% with a median DCR of 5.7 kcps at an excess bias of 3 V.

A different approach was chosen in refs. ^137,142,143^, where a 2 × (4) × 128 SPAD array, again in a standard 0.35 μm CMOS process, featured 4 sub-ns time gates during which the on-chip electronics counted the number of detected photons. This architecture also allowed the determination of the level of the residual fluorescence and DCR in addition to the Raman signal. The fill factor of each SPAD was 23%, the minimum time gate width was 80 ps, and its variation along the spectral axis (timing skew) was ±17.5 ps for a nominal width of 100 ps; the effect of inhomogeneities of this order in the samples of interest for biophotonics applications (featuring ns- and sub-ns scale fluorescence backgrounds), although small, was also discussed. The overall instrument response function (IRF) was reported to be 250 ps, and Raman spectra of several drugs of interest were acquired with a ps pulsed laser at 532 nm^144^, enabling the authors to reveal previously unseen Raman spectral features. The same underlying standard CMOS technology was used to design a gated 4 × 128 SPAD array featuring an additional 512-channel 3-bit flash TDC^145^, which allowed high timing resolution measurements (78 ps, 10 ps standard deviation in the first four bins) at the beginning of its 3.5 ns measurement range. Several SPADs were again employed at a given spectral point, to reduce the impact of noisy pixels.

Further inhomogeneity studies have been carried out by the same group, including a study employing a 256 × 16 SPAD test array with two on-chip TDCs, leading to a detailed investigation of the effect of the DCR and PDE on one hand, and gate length variations and temporal skews on the other. The latter can indeed have an important role, with growing design complexity, on the derivation of the Raman spectrum when targeting 100 ps accuracy levels^146,147^. In general, the spectra have been found to be subject to a larger deterioration, as could be expected, when the fluorescence lifetimes and levels become shorter and higher, respectively. An efficient post-processing method relies on a calibration with a known smooth (with respect to the wavelength) fluorescence background; this approach was discussed in ref. ^148^, using a high-precision TDC (<50 ps) whose range exceeded the expected timing skew, and sampling a part of the fluorescence spectrum.

The linear 256 × 2 array by Krstajić et al.^24^, which was already mentioned in the FLIM section, has also been used for surface-enhanced Raman spectroscopy (SERS), again within the (https://www.proteus.ac.uk/) PROTEUS project. Significant hardware and software improvements of the same sensor were reported in ref. ^68^, whereas the removal of fluorescent background signals (in addition to DCR) was illustrated in ref. ^67^.

### Conclusions

SPAD arrays have a unique position in time-resolved Raman spectroscopy, offering, in a compact, all solid-state system, a high spatial resolution, integrated gating and/or photon time-stamping in the sub-nanosecond range for fluorescence background rejection. From this perspective, it is surprising that time-resolved SPAD arrays have not yet seen a more widespread use for this application. This is partly due to the sensitivity of the SPADs, which degrades rapidly towards the NIR region, limiting their applicability in further reducing the autofluorescence background by moving to longer excitation wavelengths. Developments are thus heavily focused on the improvement of the sensitivity in the red and NIR regions, and further reducing the gate length and increasing the gate uniformity for biological specimens.

## Optical tomography

Near-infra-red optical tomography (NIROT) studies the absorption and scattering of light in turbid media, e.g. biological tissue. Light that has propagated in a medium is detected at its surface. The measurement process is performed at multiple wavelengths to exploit the knowledge of the absorption and scattering properties of the constituent absorbers. This prior knowledge can then be used to make a 3D reconstruction of the distribution of these absorbers within the medium; this enables, for example, a non-invasive determination of oxygenation values, usually by working in the 650–850 nm window (enhanced tissue penetration).

The corresponding image reconstruction inverse problem is, however, ill-posed by nature, leading to a low spatial resolution (1 cm level) with existing PMT-based NIROT systems; the latter have seen little use in clinical settings, and are difficult to scale up. The spatial resolution can be improved by increasing the measured amount of information, i.e. ideally, the amount of detectors and their temporal information in the case of time-domain systems. This has led to interest in solid-state detectors and in particular SPADs^149^, either as single devices or in the form of SiPMs, potentially enabling contactless setups and optimised reconstruction algorithms.

While SiPMs can indeed be combined to form a wide-area detector^150^, separate TCSPC electronics are still required, and cost might also be an issue. Another route has, therefore, explored fully integrated CMOS SPAD arrays featuring a high PDE and timing accuracy, with thousands of detection channels. An overview of the topic is provided, for example, in refs. ^41,151,152^, which also include application examples based on the LASP 128 × 128 TCSPC array^17^. The latter employed 32 10-bit 98 ps column-based TDCs in a pulsed (time-domain) setup recording the time-of-flight information.

**Fig. 6:**
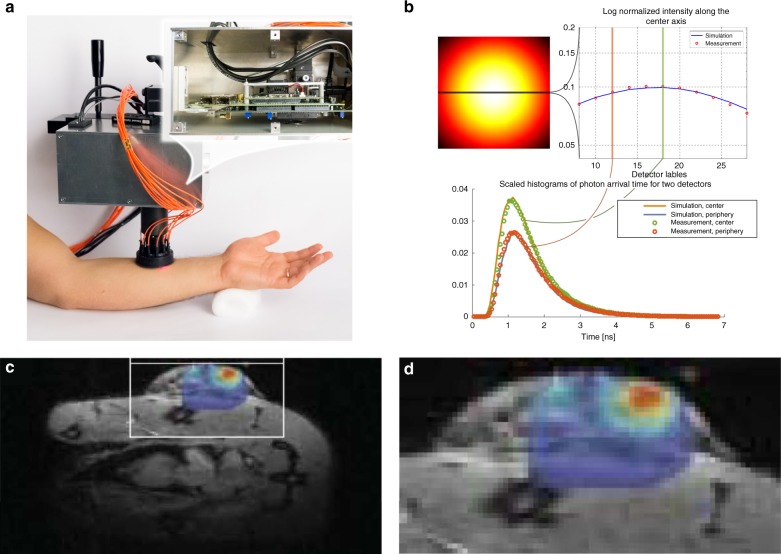
SPAD optical tomography images and applications. **a**, **b** NIROT camera system prototype and measurements versus simulation results for a phantom^154^. **c**, **d** Fluorescence molecular tomography (FMT) image as an overlap of the optical image obtained with the RadHard2 32 × 32 photon-counting sensor with the corresponding MRI image^156^. C51 cells (a colon cancer-derived cell line) have been implanted in the flank of a mouse. A clear spread in the protease activity, indicated by the significantly higher fluorescence intensity in some parts of the tumour, is shown. **c** Complete MR + FMT image, and **d** zoom of the cancer region. The images are reprinted from refs. ^154,156^

An enhanced CMOS SPAD sensor optimised for NIROT was described in refs. ^95,153^, targeted at addressing the slow acquisition time bottleneck of previous implementations, which can lead to motion artefacts and decreased patient comfort. This 32 × 32 SPAD array, called Piccolo, employed 128 TDCs, based on the concept of dynamic TDC reallocation to reduce the timing-related die area, while simultaneously reducing the probability of photon pile-up. It is implemented in a 180 nm process employing a large spectral range SPAD and coupled to a cascoded passive quenching circuit to boost the pixel’s PDE, reaching a PDP greater than 10% at 800 nm with a native fill factor of 28% at a pixel pitch of 28.5 μm. The initial results indicated a minimum image acquisition time of 3.8 s per source and wavelength pair. The sensor has been integrated into a system combining a supercontinuum laser and an acousto-optical filter for a multi-wavelength excitation, and a fibre switch to obtain up to 24 source positions^154^ (Fig. 6a); phantom measurements are in good agreement with simulations (Fig. 6b). Future implementations could employ a SPAD gating feature to reduce the impact of the early backscattered photons in certain illumination geometries^155^.

FMT (fluorescence molecular tomography) relies on (exogenous) fluorescent molecules to enable the reconstruction of their concentration distribution in 3D; FMT can be applied to small animals for pre-clinical cancer research, and possibly over time to track dynamic phenomena. A hybrid MRI-FMT imaging system was demonstrated in refs. ^156,157^, with the imaging component based on the RadHard2 (ref. ^107^) 32 × 32 photon-counting SPAD array. The results are shown in Fig. 6, where the overlap of the MRI and FMT images of a mouse tumour is displayed in Fig. 6c and enlarged in Fig. 6d. The corresponding findings were later confirmed by histological analysis of the tumour.

Finally, it is worthwhile to mention the possibility of using the exquisite timing capabilities and spatial resolution of SPAD arrays to detect ballistic and snake photons that have travelled through tissue, such as demonstrated in ref. ^158^. The MEGAFRAME32 sensor was employed to locate the distal-end of a fibre-optic device deep in tissue, with cm resolution, within clinically relevant settings and in chest and lung models on one hand, and through the entire thickness of a human torso on the other.

### Conclusions

Recent, fully integrated SPAD imagers developed for NIROT show a substantial increase in the array resolution and photon throughput due to on-chip time-stamping and histogram generation, over and beyond what can be obtained by composing arrays of single devices or SiPMs. This will likely lead to improved spatial resolution in the reconstructed image, albeit at the expense of increased system complexity. Further developments focus on an increase in the sensitivity in the red and NIR regions on one hand, and an increase in the dynamic range by time-domain gating on the other. The latter will enable to measure faster and deeper in tissue (potentially 2× with respect to a non-gated scenario).

## Other biophotonics applications and sensor concepts

### PET

Among several other biophotonics applications, which have been the subject of investigations with SPAD arrays, the nuclear medicine domain, and PET in particular, have a prominent role. Its peculiar architectural implications have already been analysed at the end of the Array architecture section, and are particularly interesting because they basically span the full pixel granularity range, from small (mm-sized) silicon photomultipliers (SiPMs) where all SPADs are connected together to provide a common output, to imagers with individually addressable SPAD pixels. The former, which are already employed in analogue form in top-end PET systems, are being revisited, e.g. monolithically with the addition of on-chip TDCs, while keeping backward compatibility^159^, or by coupling an off-the-shelf analogue SiPM array to an FPGA-based board to enable advanced timing functionality with relatively simple hardware modifications^160^. The latter are being extended in 3D, as will be described in the corresponding section below.

### Q-LSRM

On the more future-oriented side, the SPAD’s spatial and time-resolved capabilities are being investigated to enable quantum-based super-resolution microscopy (Q-LSRM). In principle, using a quantum correlated *N*-photon state combined with an optical centroid measurement (OCM) allows a resolution enhancement of 1/*N*. Apart from the significant challenges on the source side, such an approach calls for a high detection efficiency, timing resolution and frame rates on the detection side, while minimising the crosstalk and DCR. An early implementation of a monolithic 4 × 4 G^(2)^ SPAD imager was reported in refs. ^161,162^, which aimed at resolving second-order intensity correlations. More recently, the SUPERTWIN project started looking at all solid-state technologies for the generation and recording of entangled photons, targeting a resolution of 40 nm. The much larger and advanced SPADnet1 SPAD array, originally designed for PET applications, was employed in a first proof-of-principle experiment to detect spatially entangled photon pairs, generated by spontaneous parametric down-conversion (SPDC) in a non-linear crystal pumped with an intense laser beam^163,164^. The collected experience from this approach allowed to proceed with the design of an ad hoc detector, incorporating on-chip features to increase the duty cycle (e.g. to avoid reading empty frames). A 32 × 32 SPAD array was manufactured in a 150 nm CMOS process, allowing 50 ns long observation windows at up to 800 kHz to measure the 1st (G^(1)^) and 2nd (G^(2)^) order correlation functions, and the array was experimentally characterised again with a source of entangled photon pairs^36^.

### Alternatives to monolithic approaches

A sensor concept that is an alternative to the monolithic approaches described so far, built around the use of “reconfigurable pixels”, was already hinted at the end of the “Read-out architecture” section. The underlying idea is to be able to reconfigure the main data processing features as a function of the specific application needs, as is the case with the LinoSPAD 256 × 1 linear array. This system is designed in such a way that the sensor layer hosting the actual SPADs (with a fill factor of 43%) is decoupled from the data processing features, which are embedded in a companion FPGA. The latter contains core processing blocks such as a 64-element parallel TDC array^54,55^ for time-correlated applications, whereas others are reconfigurable and can in principle be combined in a modular fashion. Another advantage of this approach resides in the possible combination of a sensing-optimised layer with a more advanced processing tier (e.g. 40 or 28 nm) and the possibility of exchanging one or the other as technology evolves or new firmware is designed.

### 3D-stacking

**Fig. 7:**
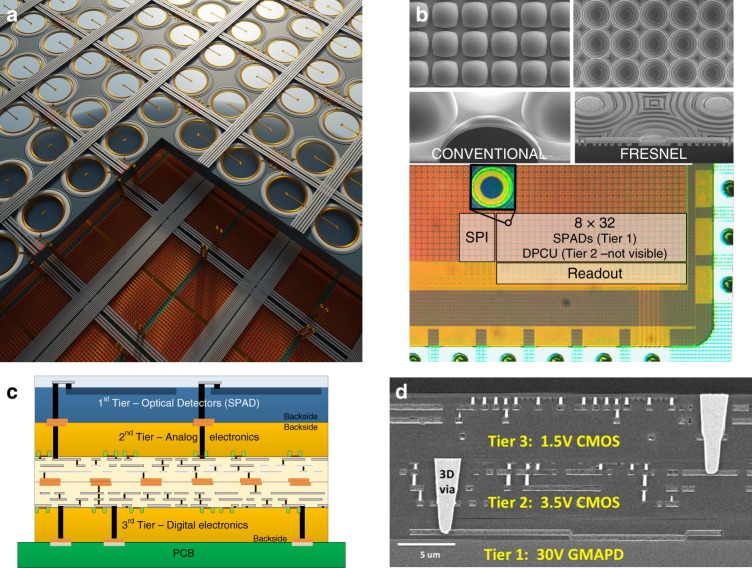
Recent SPAD concepts for imagers revolve around 3D integration, possibly combined with microlenses to further maximise the fill factor. **a** A 3D integration concept image, **b** a two-tier implementation with additional microlenses^179^ and **c**, **d** cross-sections of different imagers using three tiers^165,172^. Frontside illumination is used in **c**, whereas backside illumination is used in **b** and **d**. The images **b**–**d** are reprinted from refs. ^165,172,179^

The combination of a top sensor layer with a bottom (likely all-digital CMOS) control and processing layer, each optimised for the respective function, can also be achieved with 3D-stacking techniques (see Fig. 7a for a concept image); these techniques are progressively becoming accessible to a larger user community and benefit from the developments in consumer markets (e.g. cameras for mobile phone applications), where significant resources are available. Such an approach could potentially enable a high PDE, low DCR and reduced jitter and afterpulsing, while adding advanced functionality and low power consumption due to the use of smaller technology nodes in the bottom-tier. In addition, the use of compound semiconductors for the top layer, such as InGaAs, could open up additional imaging windows in the NIR and even mid-wave IR regions, leading to enhanced tissue penetration (see, for example, Fig. 7d and refs. ^165,166^ for a summary of extensive work in this direction by MIT’s Lincoln Laboratory). Another route to an enhanced NIR sensitivity with silicon-based platforms is backside illumination (BSI), similar to what has already been implemented in most high-end CMOS consumer imagers, whereby the substrate of the top wafer needs to be thinned down to only a few micrometres; this can be combined with thicker active volumes to counteract the reduced absorption of silicon in the NIR region. In general, the cost associated with moving to 3D is a higher design complexity, challenging 3D (wafer level) bonding techniques and the corresponding development costs.

Early work towards CMOS 3D IC SPAD-based imagers included a proof-of-concept 400 × 1 linear array specifically designed with NIROT applications in mind^152,167^, which combined 400 SPADs on the top tier with 100 TDCs (50 ps LSB) on the bottom layer in a 130 nm process. In parallel, the use of silicon-on-insulator (SOI) was explored in refs. ^168,169^, with the aim of fabricating an array of 32 × 32 custom (BackSPAD) photodetectors in a 3-μm-thick SOI film of an SOI wafer (0.35-μm feature size). The latter was then flipped and wafer bonded to a second 0.35 μm CMOS wafer with the ancillary electronics. A large body of work is available from Sherbrooke on 3D approaches specifically dedicated to PET, with the aim of achieving a possible “ultimate” one-to-one coupling between each pixel and its corresponding quenching and timing electronics^170,171^. An important part of the effort was dedicated, not surprisingly, to the 3D integration process itself, with proofs of principle along the way such as^172^ detailing the design of the top tier 0.8-μm high-voltage CMOS SPADs in a frontside illumination approach (FSI, see Fig. 7c), and the corresponding challenges in terms of connections (use of through silicon vias).

More recently, several papers have reported work exploiting advanced commercially available 3D BSI technologies. The work was in part driven by the need for optimised, small-pitch SPAD arrays with enhanced red and NIR sensitivity for consumer applications, such as light detection and ranging (LiDAR); this has led to SPAD implementations that were unthinkable in such technology nodes just a few years ago, opening the door to a potential extension to megapixel arrays. In ref. ^173^, for example, the pixel pitch was reduced to 7.83 μm, leading to a 128 × 120 time-gated prototype combining an imaging specific 65 nm top tier with a 40 nm bottom-tier employing 1-to-1 hybrid bond connections. A different implementation in the same 3D IC CMOS process was reported in^174^, achieving a higher PDE due to cascoded passive quenching and active recharging, while still being compatible with the transistor operating voltage regimes of such highly scaled technologies. The example in Fig. 7b employs a 45 nm sensor tier and a 65 nm low-power processing tier, adding microlenses for fill factor enhancement. Very low afterpulsing was obtained with a very short 8 ns dead time. Finally, the top tier was further scaled down to 45 nm in ref. ^175^, while further improving the peak PDP and reducing the jitter and DCR. The DCR is in general 2–3 orders of magnitude higher than the best FSI technologies, whereas the PDP, which is typically very low in the blue region for BSI implementations, has improved to above 30% in the red region, with fill factors in excess of 50%.

## Summary

**Fig. 8:**
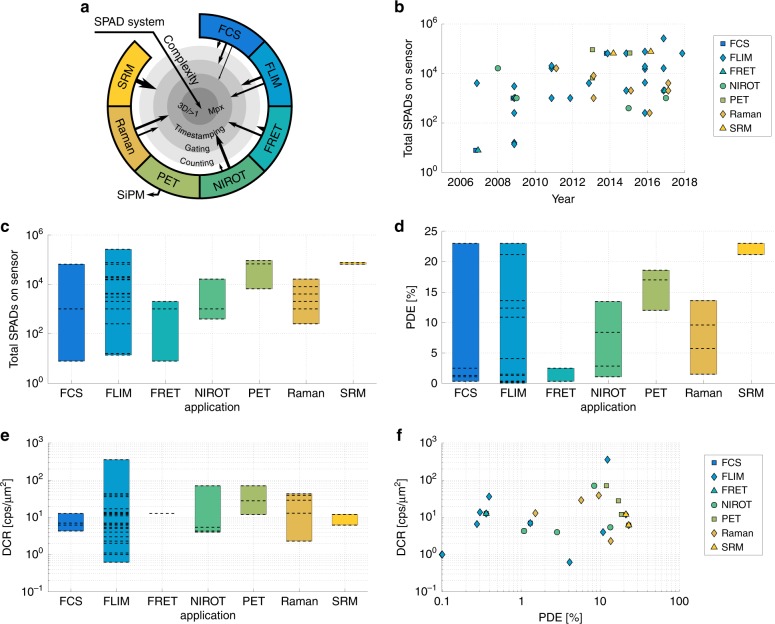
SPAD system complexity vs. biophotonics applications and evolution of representative SPAD sensor figures of merit. **a** Schematic overview of the SPAD-based system complexity, in terms of key functionalities (counting/gating/time-stamping) versus the main biophotonics applications. **b**–**f** Overview of the representative SPAD sensor figures of merit as a function of the main target applications, based on data from Table 2: **b** total number of SPADs (corresponding to the effective spatial resolution in the imagers) versus time; **c**–**e** total number of SPADs, PDE and DCR per unit area grouped based on the application types (dashed lines: individual sensors, top/bottom of each box: maximum/minimum); and **f** the DCR per unit area versus the PDE

Figure 8a provides a schematic overview of how the key functionalities of the SPAD-based imagers reviewed in this paper are related to the main biophotonics applications to which they have been applied so far. The arrows pointing towards the centre provide a qualitative indication of the relative importance of the different functionalities, whereby most applications have seen the use of at least two of the functionalities (e.g. time-stamping and gating), except localisation-based super-resolution microscopy (SRM), which has only needed photon counting so far. PET is a somewhat special case, relying on ad hoc architectures as discussed in the first paragraph of the previous section.

Figure 8b shows the distribution of the total number of SPADs over time, based on the data from Table 2, indicating a clear trend towards larger SPAD arrays over the past decade. Figure 8c–e illustrates the distribution of some of the key figures of merit (total number of SPADs, PDE and DCR per unit area), again using Table 2 as a reference, grouped based on the main biophotonics application types. Each dashed line corresponds to an individual sensor. For the total number of SPADs and the PDE, higher values are preferred, whereas the contrary is obviously true for the DCR. The size of each box is representative of the spread of the figures of merit for that specific application; the size is, for example, larger for FLIM, where the first developments took place early on, when the technology and designs were far from optimised. The box size also reflects the number of sensors that have been employed thus far; from this perspective, the size is smaller for SRM, which has only been addressed very recently.

Finally, as discussed in the SPAD section, the design of SPAD sensors entails numerous trade-offs, such as the PDE versus DCR shown in Fig. 8f. This plot indicates a move towards higher PDE values, although this is not easy to achieve while still maintaining a low DCR (an ideal sensor would be placed in the bottom right corner of this plot).

## Conclusions and outlook

In this review, we have focused on SPAD imagers in standard CMOS technologies and their biophotonics applications. Individual SPAD pixels and small arrays have evolved from a scientific curiosity 15 years ago to a range of fully integrated devices, where the key challenges have been the sensitivity, homogeneity, noise, reliability and reproducibility.

At the device level, the performance gap between SPADs fabricated in custom technologies and in standard CMOS technology has been shrinking over time, whereby CMOS can leverage 60 years of experience and investments in scalable and high-yield technologies. The corresponding improvements have been significant in terms of key parameters, such as the PDP and fill factor, with peak values in excess of 40–55%, the DCR, which has been drastically reduced to below 0.1 cps/μm^2^ in the best SPADs, and the percentage of hot pixels (just a few percent in the best technologies). The spatial resolution has also increased to a quarter megapixel for the largest formats, with megapixel arrays on the horizon. This has called for a decrease of the pixel size to well below 10 μm, with some groups targeting SPADs in deep sub-micron technology nodes (45 nm and below), not very far from their counterparts in CMOS imagers; this feat was unthinkable just a few years ago. However, achieving multi-megapixel SPAD sensors is challenging, as SPADs cannot be easily decreased in size below 3 μm (pitch) in currently available technologies (see ref. ^176^ for results on a 4 × 4 test structure). This is due to the required guard ring and the correlated reduction of the active area to zero due to edge effects. The timing accuracy was already excellent (typically 50–100 ps, with the best SPADs in the 20–30 ps range) and has, therefore, evolved in a less spectacular fashion.

At the architectural level, standard CMOS SPAD arrays have a clear advantage over CCDs/sCMOS imagers, when parallel single-photon counting and/or timing is required; standard CMOS SPAD arrays can be coupled in a flexible way to different digital blocks for data acquisition and/or processing in close proximity to the sensor, thanks to the natively digital SPAD data output. The absence of read-out noise is an important issue that is often neglected, particularly when SPAD imagers are coupled to very high speed (~100 kfps), ADC-less binary implementations, delivering continuous microsecond frames in real-time to capture fast transient phenomena. The capability of implementing an integrated, parallel nanosecond gating is also a very appealing alternative to non-all-solid-state implementations. However, such short gate windows have to be properly implemented to achieve a reasonable skew (below 150 ps) and with the corresponding steep rise and fall times. This can be challenging in large SPAD arrays due to the power consumption required by the distribution of the digital gating signals across the array.

Therefore, it is not surprising that FLIM has been explored first, together with multibeam approaches that make full use of the sensors’ native parallelism. This was then followed by a host of other time-resolved biophotonics applications, all the way to disruptive scenarios, such as quantum-based super-resolution microscopy; the latter represents a good example of a sensor architecture that has been custom-designed to meet specific requirements, for which efficient sensor alternatives are rare. It is, however, true that most SPAD imagers are still research prototypes, and only some are available commercially, e.g. from SMEs such as PhotonForce (UK) or MPD (Italy), usually as a spin-off of designs explored in academic environments. (Other commercially available SPAD arrays are usually derived from non-imaging SiPMs, e.g. the Philips Digital Photon Counting dSiPM for clinical PET, or the STMicroelectronics time-of-flight sensors^177^, aimed at ranging in the consumer market).

This is partly due to the maturity of the sensors themselves and/or of the underlying technology, and partly to the fact that the overall sensitivity, in particular in the red region, still lags behind the sensitivity of CCD/sCMOS imagers by 2–10×. This can be an issue in applications where absolute sensitivity, rather than the SNR, is important, or where the illumination power needs to be kept low to avoid sample degradation. Certain applications require a very high timing resolution, which is typical of single, highly optimised SPADs, and can tolerate longer acquisition times due to scanning. It is also true that for SPAD imagers, system integration can typically be more complex due to the high data rates and corresponding tight interconnect and firmware requirements. Finally, in certain cases SPAD designers face competition from other single-photon or established technologies.

In the future, we foresee that academic and research establishments will continue pushing the state-of-the-art in terms of the key figures of merit, targeting large-format and high-performance CMOS SPAD arrays, with industrial applications starting to appear in the medium term. Larger industrial conglomerates are more likely to emphasize high-volume applications in the mobile/consumer areas, e.g covering automotive (in particular LiDAR for advanced driver-assistance systems), point-of-care and Internet of Things (IoT) applications, possibly via smaller, dedicated units; these foundries might also propose SPADs as IP blocks in the not too distant future. The resulting consumer oriented developments are likely to follow trends similar to those that characterised high-end smartphone imagers (i.e. towards chip stacking and 3D ICs); these developments might impact in a positive way niche markets in general, and time-resolved applications of interest to the biophotonics community in particular.
