# Author Correction: Single-photon avalanche diode imagers in biophotonics: review and outlook

**DOI:** 10.1038/s41377-020-0248-5

**Published:** 2020-01-28

**Authors:** Claudio Bruschini, Harald Homulle, Ivan Michel Antolovic, Samuel Burri, Edoardo Charbon

**Affiliations:** 1 AQUA, EPFL, Neuchâtel, Switzerland; 2 AQUA, TU Delft, Delft, The Netherlands

**Keywords:** Optics and photonics, Physics

**Correction to: Light: Science & Application**

10.1038/s41377-019-0191-5 published online 18 September 2019

The authors would like, for the sake of transparency, to expand the original Conflict of interest statement (page 25) as follows:

The authors declare that there are no conflicts of interest related to this article. For the sake of transparency, the authors would like to disclose that (i) Edoardo Charbon holds the position of Chief Scientific Officer of Fastree3D, a company making LiDARs for the automotive market, and that (ii) Ivan Michel Antolovic, Claudio Bruschini and Edoardo Charbon are co-founders of Pi Imaging Technology. Both companies have not been involved with the paper drafting, and at the time of writing have no commercial interests related to this article.

